# Insights into Selenium-Modulated Amino Acids and Carbohydrates as Osmolytes Linked to Photosynthetic Efficiency in Drought-Stressed Edamame

**DOI:** 10.3390/plants15131943

**Published:** 2026-06-24

**Authors:** Masego Sekhurwane, Mpho Mafa, Zoltán Kovács, László Kaszás, Béla Kovács, Brigitta Tóth, Makoena Joyce Moloi

**Affiliations:** 1 Department of Plant Sciences, Faculty of Natural and Agricultural Sciences, Bloemfontein Campus, University of the Free State, P.O. Box 339, Bloemfontein 9300, South Africa; sekhurwanemasego@gmail.com; 2 Carbohydrates and Enzymology Laboratory (CHEM-LAB), Department of Plant Sciences, University of the Free State, P.O. Box 339, Bloemfontein 9300, South Africa; mafams@ufs.ac.za; 3 Department of Applied Plant Biology, Institute of Crop Sciences, University of Debrecen, Böszörményi Street 138, 4032 Debrecen, Hungary; kovacs.zoltan@agr.unideb.hu (Z.K.); kaszas.laszlo@agr.unideb.hu (L.K.); 4 Institute of Food Science, Faculty of Agricultural and Food Sciences and Environmental Management, University of Debrecen, Böszörményi Street 138, 4032 Debrecen, Hungary; kovacsb@agr.unideb.hu; 5 Research and Innovation Center, University of Nyíregyháza, Sóstói Str. 31/b, 4400 Nyíregyháza, Hungary

**Keywords:** drought stress, selenium, osmolyte metabolism, photosynthetic efficiency, vegetable-soybean

## Abstract

Drought impairs osmotic adjustment and photosynthetic performance in legumes; however, the role of micronutrients in modulating these responses across genotypes remains unclear. This study investigated the effects of selenium on the osmolytes and photosynthetic efficiency in two vegetable-soybean (*Glycine max* L. Merrill) cultivars differing in drought responses: UVE14 (drought-tolerant) and UVE17 (drought-susceptible). Plants were grown under well-watered (100% soil water-holding capacity, WHC) and water-limited (30% soil WHC) conditions, with or without soil-applied selenium. Free amino acids, soluble sugars, chlorophyll pigments, vegetation indices, and chlorophyll fluorescence parameters were assessed at the flowering and pod-filling stages. Under drought conditions, selenium enhanced tolerance primarily by modulating free amino acid metabolism at flowering, increasing aspartate, asparagine, glutamine, and glutamate levels, alongside improvements in chlorophyll content, canopy greenness, and PSII photochemical efficiency. These responses indicate a coordinated adjustment between nitrogen metabolism and photosynthetic function. Both cultivars benefited from selenium application, although the responses were more pronounced in the susceptible cultivar (UVE17). Selenium-induced changes in soluble sugar content were greater under well-watered conditions in both cultivars. The limited accumulation of stress-associated osmolytes, such as proline, following selenium soil drench suggests reduced cellular disruption and mitigation of drought-induced stress. These findings highlight selenium as a context-dependent modulator of drought resilience and emphasize cultivar- and developmental stage-specific effects.

## 1. Introduction

Drought, intensified by anthropogenic climate change, poses a major challenge to global agriculture [1]. Global food insecurity is predicted to increase by 60% as the world population approaches nine billion by 2050 [2]. In regions with rapidly growing populations, such as sub-Saharan Africa, drought has caused crop losses of up to 50%, while major crops, including soybean, have experienced yield reductions of up to 40% [3,4]. Vegetable-soybean, also known as edamame (*Glycine max* L. Merrill), is a drought-sensitive legume whose growth and productivity are severely constrained under water-deficit conditions [5]. Its high protein and oil contents, exceeding 40% and 20%, respectively, make it an important crop for food security and sustainable development [6]. Drought stress, especially during the reproductive stages, disrupts key physiological processes, including photosynthesis, resulting in reduced growth and yield [7]. For example, drought drastically inhibited growth and yield in *Tulipa edulis* [8], and similar reductions have been reported in edamame [9].

Among the physiological processes affected, photosynthesis is particularly sensitive to water deficit. In tomato seedlings, drought reduced chlorophyll content, the maximum quantum efficiency of photosystem (PS) II (represented by the ratio of variable fluorescence to maximum fluorescence, Fv/Fm), and the total performance index (PI_total_) [10], while similar declines in Fv/Fm were observed in *Lonicera caerulea* [11]. Water stress reduced growth, yield, and chlorophyll pigments in quinoa [12] and decreased chlorophyll *a* and *b* contents in drought-susceptible edamame [13]. Reduced photosynthetic efficiency limits the production of photoassimilates such as sucrose [14], which, together with other soluble sugars, including glucose, fructose, and trehalose, contribute to osmotic adjustment and the maintenance of leaf water status under drought conditions [13,15]. As major products of photosynthesis, soluble sugars serve as primary sources of carbon and energy in plants [16]. Their accumulation reflects both photosynthetic performance and metabolic activity, including carbohydrate oxidation through glycolysis and the Krebs cycle. During drought stress, sucrose also functions as an osmolyte and may serve as a precursor for the synthesis of other protective sugars, such as trehalose, raffinose, and fructans, which contribute to stress tolerance [17].

The accumulation of osmolytes, including soluble sugars, glycine betaine, proline, and other free amino acids, supports cellular structure and stress tolerance [18]. Although not directly produced by photosynthesis, free amino acids such as proline, alanine, arginine, glycine, glutamate, and asparagine serve as precursors for many osmolytes and help protect cell membranes [19]. Glutamate, in particular, is a precursor for both proline biosynthesis [20] and the tetrapyrrole pathway involved in chlorophyll synthesis [21], and it also promotes shoot growth [20]. Aspartate enhances disease resistance, while arginine increases tolerance to heat and drought stress and contributes to chlorophyll biosynthesis. Glycine promotes photosynthesis and chlorophyll formation, whereas proline scavenges reactive oxygen species and enhances stress tolerance [20]. Under drought conditions, rice accumulates increased levels of proline [22], and other studies have suggested that proline contributes to stress tolerance through its antioxidant activity and osmoprotective functions [23]. Edamame accumulates soluble sugars and starch under drought and combined heat and drought stress, thereby contributing to osmotic adjustment [16,24]. However, the roles of amino acids, either individually or in combination with carbohydrates, in osmotic adjustment and drought tolerance in edamame remain poorly understood. In drought-susceptible plants, delayed or insufficient osmolyte accumulation may impair stress protection mechanisms [25].

To mitigate these effects, selenium, a beneficial trace element, has been explored as a regulator of plant stress responses [26,27]. Selenium can enhance photosynthetic efficiency, protect photosystems I and II, promote osmolyte accumulation, and improve water-use efficiency under stress [28]. Foliar application of selenium increased the relative water content and osmolyte accumulation, thereby enhancing photosynthesis efficiency and growth in onion, wheat, and strawberry plants [29,30,31,32]. In vegetable-soybean, foliar selenium application at the vegetative stage enhanced antioxidative enzyme activity, although improvements in yield were limited [33]. Recent evidence indicates that soil-applied selenium effectively modulates physiological and biochemical responses, improves plant growth, and enhances yield-related traits under drought conditions [34].

Despite these advances, the effects of selenium soil drenching on amino acid and soluble sugar metabolism under drought conditions in vegetable-soybean remain unclear. Therefore, this study investigated the effect of soil-applied selenium on osmolyte metabolism and photosynthetic efficiency in drought-tolerant and drought-susceptible vegetable-soybean cultivars subjected to drought stress at the flowering and pod-filling stages. It further explores the potential links between selenium-induced metabolic changes and photosynthetic performance. By integrating amino acid and carbohydrate profiles with chlorophyll traits, vegetation indices, chlorophyll fluorescence parameters, and correlation analyses, this study aimed to elucidate how selenium modulates the coordination between metabolism and photosynthesis under drought stress across cultivars and developmental stages.

## 2. Results

The significance of main effects and interactions was assessed using three-way analysis of variance (ANOVA), with the full ANOVA results presented in Supplementary Tables S1 and S2. To facilitate interpretation, the Section 2 is organized according to the hierarchical structure of the ANOVA. Variables exhibiting significant cultivar × water level × selenium (C × W × Se) interactions are presented first, followed by variables showing significant two-way interactions in the absence of significant three-way interactions. For variables with no significant interactions, only the relevant main effects are reported. This approach was applied to amino acids, soluble sugars, chlorophyll traits, and photosynthetic parameters measured in the vegetable-soybean cultivars UVE14 and UVE17 at the flowering and pod-filling stages.

### 2.1. Amino Acids

Four amino acids (Asparagine + Aspartate, represented as Asx; Glutamate + Glutamine, represented as Glx) exhibited significant C × W × Se interactions (Supplementary Tables S1 and S2). The corresponding treatment means are presented in Table 1 and discussed below.

At flowering, drought stress elicited contrasting cultivar responses, increasing Asx by 138% in UVE14 but decreasing it by 42% in UVE17 relative to the corresponding well-watered plants without selenium. Selenium application under drought conditions increased Asx by 93% in UVE17 compared with drought-stressed plants that did not receive selenium. Under well-watered conditions, selenium also enhanced Asx accumulation in UVE14, resulting in a 98% increase relative to the corresponding untreated plants. At pod-filling, selenium application reduced Asx by 95% in well-watered UVE14 plants compared with the untreated control. A similar cultivar-specific response was observed for glutamate and glutamine (Glx) at flowering. Drought stress increased Glx by 48% in UVE14 but decreased it by 48% in UVE17 relative to the corresponding well-watered plants without selenium. Selenium application under drought conditions increased Glx by 78% in UVE17 but decreased it by 25% in UVE14 compared with the respective drought-stressed plants that did not receive selenium. Under well-watered conditions, selenium increased Glx in both cultivars relative to the respective untreated control. At pod-filling, selenium application reduced Glx by 94% in well-watered UVE14 plants compared with the corresponding untreated control.

**Figure 1:**
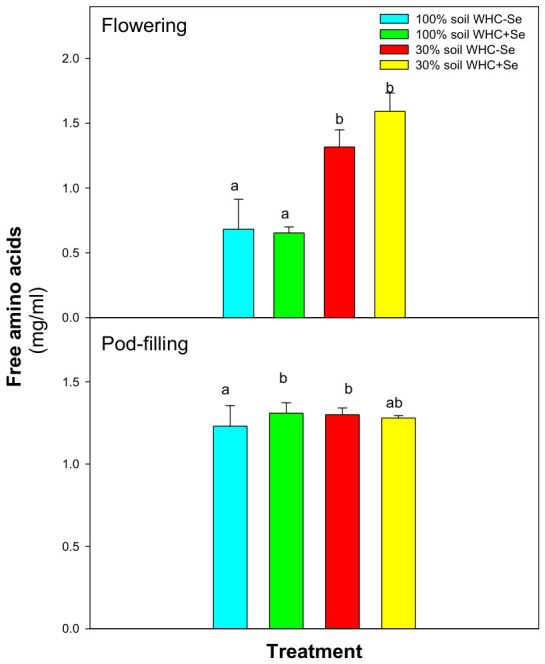
Free amino acid (FAA) accumulation in the vegetable-soybean cultivars UVE14 (drought-tolerant) and UVE17 (drought-susceptible) grown under well-watered (100% soil water-holding capacity, WHC) and drought (30% soil WHC) conditions, with and without selenium application, at the flowering and pod-filling stages. +Se: selenium application; −Se: no selenium application. Values represent means ± SE (n = 3). Fisher’s LSD post hoc test was used to determine significant differences among treatment means (*p* < 0.05). Treatments with different letters are significantly different. Treatments sharing one or more letters are not statistically different.

Total free amino acids (FAA) exhibited a significant water × selenium (W × Se) interaction at flowering in the absence of a significant C × W × Se interaction (Supplementary Tables S1 and S2). Selenium application enhanced FAA accumulation under drought stress, resulting in a 27% increase relative to drought-stressed plants that did not receive selenium. In contrast, selenium had no significant effect on FAA accumulation under well-watered conditions (Figure 1).

For traits exhibiting a significant main effect of water treatment, drought significantly reduced the concentrations of arginine (41%), alanine (17%), and proline (43%) at flowering, with no significant effects of selenium application or cultivar (Table 2).

### 2.2. Soluble Sugars

**Figure 2:**
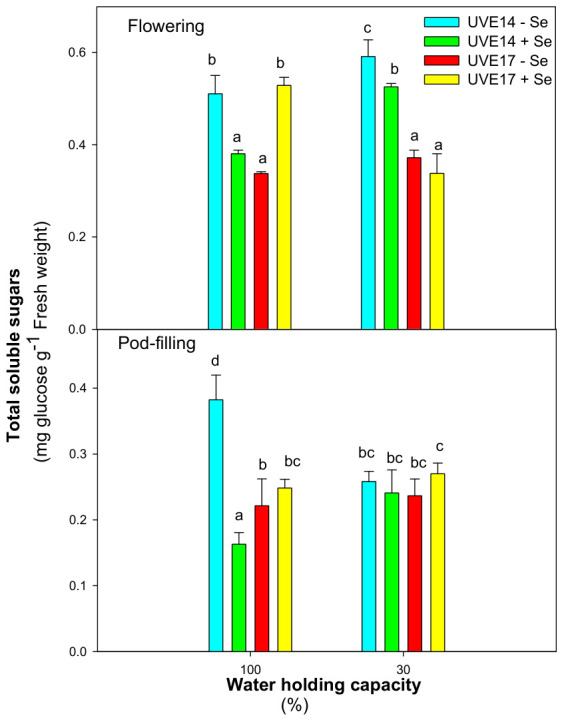
Total soluble sugar accumulation (TSS) for vegetable-soybean cultivars UVE14 (drought-tolerant cultivar) and UVE17 (drought-susceptible cultivar) grown under well-watered (100% soil water-holding capacity, WHC) and drought (30% soil WHC) conditions, with and without selenium application, at the flowering and pod-filling stages. +Se: selenium application; −Se: no selenium application. Values represent means ± SE (n = 3). Fisher’s LSD post hoc test was used to determine significant differences between treatment means (*p* < 0.05). Treatments with different letters are significantly different. Treatments sharing one or more letters are not statistically different.

**Figure 3:**
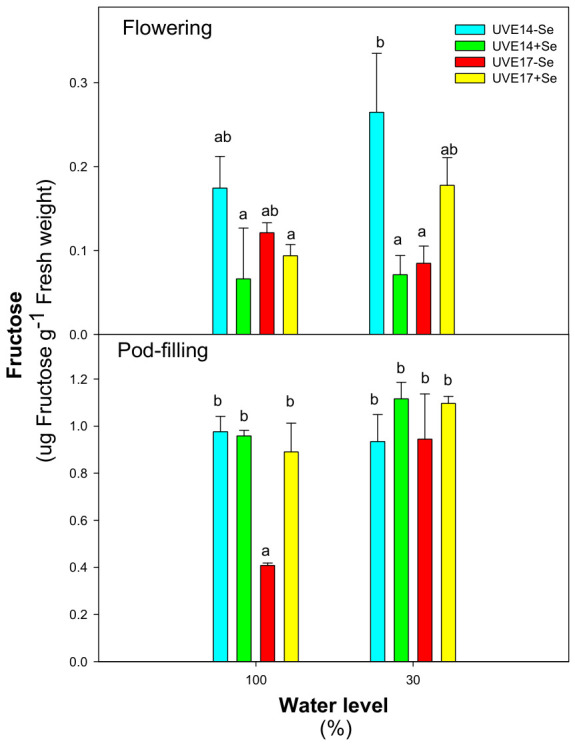
Fructose content in the vegetable-soybean cultivars UVE14 (drought-tolerant) and UVE17 (drought-susceptible) grown under well-watered (100% soil water-holding capacity, WHC) and drought (30% soil WHC) conditions, with and without selenium application, at the flowering and pod-filling stages. +Se: selenium application; −Se: no selenium application. Values represent means ± SE (n = 3). Fisher’s LSD post hoc test was used to determine the significance of differences between the treatment means (*p* < 0.05). Treatments with different letters are significantly different. Treatments sharing one or more letters are not statistically different.

**Figure 4:**
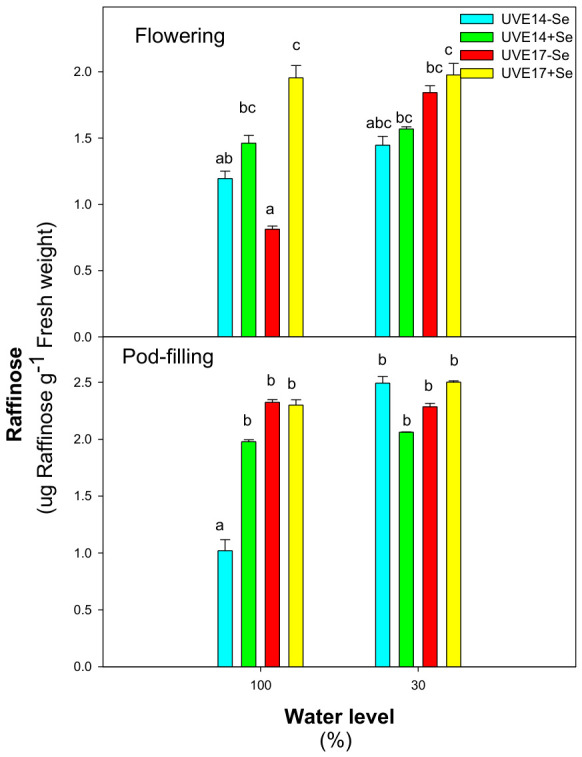
Raffinose content in the vegetable-soybean cultivars UVE14 (drought-tolerant) and UVE17 (drought-susceptible) grown under well-watered (100% soil water-holding capacity, WHC) and drought (30% soil WHC) conditions, with and without selenium application, at the flowering and pod-filling stages. +Se: selenium application; −Se: no selenium application. Values represent t means ± SE (n = 3). Fisher’s LSD post hoc test was used to determine the significance of differences between the treatment means (*p* < 0.05). Treatments with different letters are significantly different. Treatments sharing one or more letters are not statistically different.

Significant cultivar × water × selenium (C × W × Se) interactions were observed for total soluble sugars (TSS), fructose, and raffinose (Supplementary Tables S1 and S2). The corresponding treatment means are presented in Figure 2, Figure 3 and Figure 4. At flowering, drought stress increased TSS in UVE14 (16%) but had no significant effect in UVE17 relative to the corresponding well-watered plants without selenium. Under well-watered conditions, selenium application increased TSS in UVE17 (57%) but reduced it by 25% in UVE14 compared with the respective untreated controls. Under drought conditions, selenium did not significantly affect TSS accumulation in UVE17 but reduced it in UVE14 by 11% compared with drought-stressed plants that did not receive selenium. At pod-filling, drought stress selectively reduced TSS accumulation by 32% in UVE14 compared to the corresponding well-watered plants without selenium. Under these conditions, selenium application had no significant effect on either cultivar (Figure 2).

The effects of selenium on fructose content in two vegetable-soybean cultivars under drought conditions are shown in Figure 3. Drought stress had no significant effect on fructose accumulation at the flowering stage. Under well-watered conditions, selenium application did not significantly affect fructose content in either cultivar. However, under drought conditions, selenium application significantly decreased fructose accumulation in UVE14 by 73% compared with drought-stressed plants that did not receive selenium. At pod-filling, drought stress had no significant effect on fructose accumulation in UVE14. However, the UVE17 showed a significant increase (131%) in fructose accumulation under drought stress compared to the corresponding well-watered treatment without selenium. Under well-watered conditions, selenium application increased fructose accumulation in UVE17 by 118% compared with the corresponding untreated control.

The effects of selenium soil drenching on raffinose content in the two vegetable-soybean cultivars under drought conditions are represented in Figure 4. At flowering, selenium application under drought conditions had no significant effect on raffinose accumulation in either cultivar, although UVE17 showed a 7% increase. Under well-watered conditions at this stage, selenium had no significant effect on raffinose content in UVE14 but significantly increased it by 140% in UVE17 compared to the corresponding untreated control. At the pod-filling stage, drought stress alone increased the raffinose accumulation in UVE14 by 94% compared with the corresponding well-watered plants without selenium. Under well-watered conditions, selenium application selectively increased raffinose accumulation in UVE14 by 93% relative to the untreated control. Under drought conditions at this stage, selenium had no significant effect on raffinose accumulation in either cultivar.

**Figure 5:**
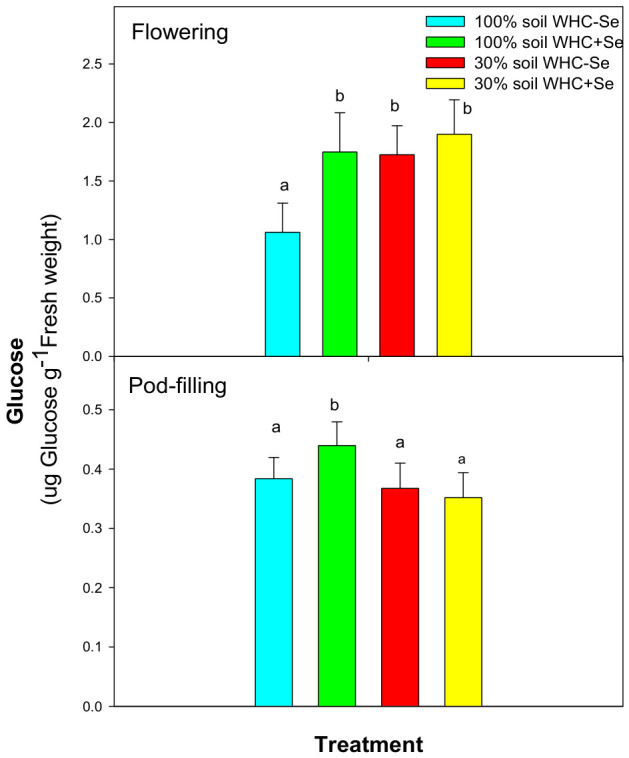
Glucose content in the vegetable-soybean cultivars UVE14 (drought-tolerant) and UVE17 (drought-susceptible) grown under well-watered (100% soil water-holding capacity, WHC) and drought (30% soil WHC) conditions, with and without selenium application, at the flowering and pod-filling stages. +Se: selenium application; −Se: no selenium application. Values represent means ± SE (n = 3). Fisher’s LSD post hoc test was used to determine the significance of differences between the treatment means (*p* < 0.05). Treatments with different letters are significantly different. Treatments sharing one or more letters are not statistically different.

Glucose exhibited a significant W × Se interaction (Supplementary Tables S1 and S2). Under well-watered conditions, selenium application increased glucose concentrations by 65% at flowering and by 16% at pod-filling relative to the corresponding untreated controls, whereas no significant selenium effect was observed under drought conditions (Figure 5).

**Figure 6:**
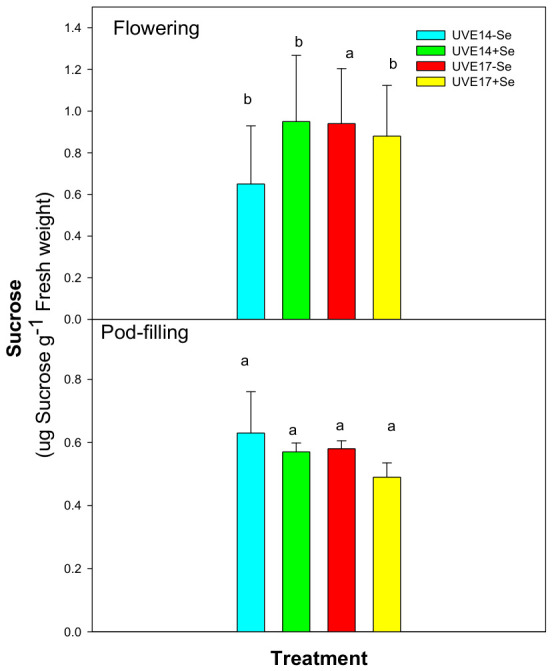
Sucrose content in the vegetable-soybean cultivars differing in drought tolerance (UVE14, drought-tolerant; UVE17, drought-susceptible) with and without selenium application, at the flowering and pod-filling stages. +Se: selenium application; −Se: no selenium application. Values represent means ± SE (n = 3). Fisher’s LSD post hoc test was used to determine the significance of differences between the treatment means (*p* < 0.05). Treatments with different letters are significantly different. Treatments sharing one or more letters are not statistically different.

Sucrose was the only soluble sugar to exhibit a significant cultivar × selenium (C × Se) interaction (Supplementary Table S1). Selenium application increased sucrose concentration in UVE14 by 46%, whereas no significant response was observed in UVE17 (Figure 6).

### 2.3. Photosynthetic Traits, Pigments, and Water Status

Several photosynthetic traits exhibited significant C × W × Se interactions (Supplementary Tables S1 and S2). The corresponding treatment means are presented in Table 3. At the flowering stage, selenium application elicited cultivar-dependent responses in pigment-related indices. Under well-watered conditions, selenium increased the chlorophyll index (CCI) by 16% and SPAD values by 8% in UVE14, whereas under drought stress, it increased CCI by 16% and SPAD values by 11% in UVE17. In contrast, selenium reduced CCI by 14% and SPAD values by 6% in drought-stressed UVE14 plants. Similarly, NDVI responses were cultivar- and water-dependent, decreasing by 2% in well-watered UVE17 plants but increasing by 2% under drought conditions. Among the chlorophyll fluorescence parameters, selenium drenching increased Fv/Fm by 3% in drought-stressed UVE17. ABS/RC was significantly reduced following selenium application in well-watered UVE14 plants (14%), drought-stressed UVE14 plants (19%), and drought-stressed UVE17 plants (14%). In contrast, selenium consistently enhanced photosynthetic performance indices. PI_abs_ increased significantly in both cultivars under well-watered conditions and under drought stress in UVE14 (62%) and UVE17 (54%). Similarly, PI_total_ increased in both cultivars under well-watered conditions, whereas under drought stress, a significant increase was observed only in UVE17 (59%). Selenium also significantly increased Chl *a* content by 30%, but only in UVE17 grown under 100% soil WHC.

During pod-filling, pigment-related traits continued to exhibit cultivar-dependent responses to selenium application. Selenium increased CCI by 16% in well-watered UVE14 and by 13% in drought-stressed UVE17 plants, whereas decreases of 10% and 12% were observed in well-watered UVE17 and drought-stressed UVE14, respectively. Similarly, NDVI decreased by 2% in well-watered UVE17, but increased by 1.5% under drought stress. In UVE14, selenium soil drench increased SPAD by 2.4% under well-watered but reduced under drought conditions. In contrast, SPAD decreased in well-watered UVE17 plants but increased by 3.6% under drought stress. Selenium also increased Chl *a* content in all treatment combinations except drought-stressed UVE17 plants. For chlorophyll fluorescence traits, selenium increased Fv/Fm by 4.1% in both cultivars under well-watered conditions. ABS/RC decreased following selenium application in well-watered UVE14 (22%) and UVE17 (21%) plants, whereas a 21% increase was observed in drought-stressed UVE14 plants. Selenium treatment markedly increased PI_abs_ under well-watered conditions, with increases of 231% and 220% in UVE14 and UVE17, respectively, while under drought stress, a significant increase was observed only in UVE14 (22%). Similarly, PI_total_ increased by 57% in well-watered UVE17 plants but decreased by 14% under drought conditions.

Among the photosynthetic traits that did not exhibit significant C × W × Se interactions, PRI, DIo/RC, TRo/RC, and ETo/RC showed significant W × Se interactions and are presented in Table 4. At flowering, PRI decreased significantly by 27% when selenium was applied under drought stress. DIo/RC decreased significantly by 30% and 13% following selenium application under well-watered and drought conditions, respectively. Similarly, TRo/RC decreased significantly by 23% and 6% under well-watered and drought conditions, respectively. In contrast, ETo/RC increased significantly following selenium application, by 26% under well-watered conditions and by 13% after under drought stress. At pod-filling, selenium application significantly reduced DIo/RC by 17% under well-watered conditions and by 22% under drought stress. TRo/RC also decreased significantly by 18% under well-watered conditions. In contrast to the flowering stage, ETo/RC decreased significantly at the pod-filling stage, by 9% under well-watered conditions and by 20% under drought stress.

Chlorophyll *b* (Chl *b*) and total chlorophyll exhibited significant C × Se interactions (Table 5). At the flowering stage, selenium soil drenching significantly reduced Chl *b* content in UVE14 by 29% and increased it in UVE17 by 53%. Selenium soil drenching significantly enhanced total chlorophyll content by 10% in cultivar UVE14. At the pod-filling stage, selenium significantly increased Chl *b* and total chlorophyll contents in UVE17 by 40% and 29%, respectively.

**Figure 7:**
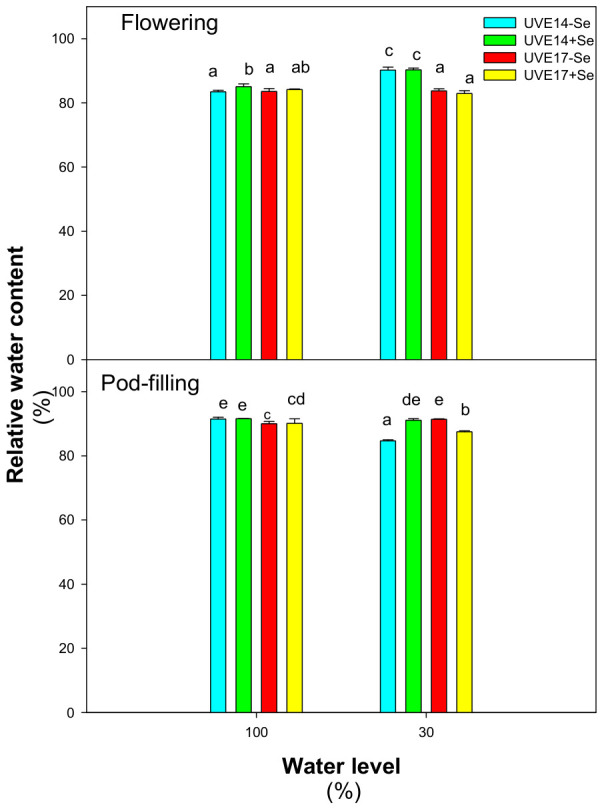
Relative water content (RWC) in the vegetable-soybean cultivars UVE14 (drought-tolerant) and UVE17 (drought-susceptible) grown under well-watered (100% soil water-holding capacity, WHC) and drought (30% soil WHC) conditions, with and without selenium application, at the flowering and pod-filling stages. +Se: selenium application; −Se: no selenium application. Values represent means ± SE (n = 3). Fisher’s LSD post hoc test was used to determine the significance of differences between the treatment means (*p* < 0.05). Treatments with different letters are significantly different. Treatments sharing one or more letters are not statistically different.

Figure 7 shows the effect of selenium soil drench on the relative water content (RWC) of two vegetable-soybean cultivars (UVE14 and UVE17) at the flowering and pod-filling stages under drought stress. At the flowering stage, selenium soil drench significantly increased the RWC of UVE14 (2%) under well-watered conditions. However, at the pod-filling stage, no significant differences were observed for well-watered conditions. In contrast, under drought stress, selenium soil drenching significantly increased RWC in UVE14 by 8% but decreased it in UVE17 by 4%.

Table 6 presents the correlation between photosynthetic efficiency and compatible solute accumulation in the vegetable-soybean cultivar UVE17 at the flowering stage under drought stress conditions. The results showed significant negative correlations between total soluble sugars and both CCI (*p* ≤ 0.01) and SPAD (*p* ≤ 0.05). Glucose was positively correlated with RWC (*p* ≤ 0.05), while sucrose content showed a significant positive correlation with CCI (*p* ≤ 0.05). Fructose content was positively correlated with PI_abs_ (*p* ≤ 0.05) but negatively correlated with CCI (*p* ≤ 0.01) and SPAD (*p* ≤ 0.05). Trehalose and raffinose showed no significant correlations with any of the measured photosynthetic parameters. However, free amino acid (FAA) content showed significant positive correlations with DIo/RC and CCI (*p* ≤ 0.05), as well as with NDVI and SPAD (*p* ≤ 0.01). FAA was negatively correlated with PRI and PI_abs_ (*p* ≤ 0.05). Arginine showed significant negative correlations with Chl *a* content (*p* ≤ 0.01) and PI_total_ (*p* ≤ 0.05). Glycine was also negatively correlated with PI_total_ and Chl *a* content (both *p* ≤ 0.01). Alanine and proline showed a significant negative correlation with Chl *a* content and PI_total_ (*p* ≤ 0.05). Glx showed a significant positive correlation with total chlorophyll content (*p* ≤ 0.05).

The correlation between photosynthetic efficiency and osmolyte accumulation in the vegetable-soybean cultivar UVE17 at the pod-filling stages under drought stress is shown in Table 7. The results showed a significant negative correlation between total chlorophyll content and CCI (*p* ≤ 0.01). Glucose was positively correlated with PRI (*p* ≤ 0.05) but negatively correlated with Chl *b* (*p* ≤ 0.01) and Tot Chl (*p* ≤ 0.05) content. Sucrose content showed no significant correlations with the measured photosynthetic parameters. Fructose content was positively correlated with total chlorophyll content, NDVI, PI_total_, ETo/RC (*p* ≤ 0.01), as well as Fv/Fm, PI_abs_, Chl *a,* and total chlorophyll content (*p* ≤ 0.05). In contrast, fructose showed significant negative correlations with ABS/RC and TRo/RC (*p* ≤ 0.01). Asx showed significant positive correlations with DIo/RC (*p* ≤ 0.01) and TRo/RC (*p* ≤ 0.05). Arginine was positively correlated with Fv/Fm (*p* ≤ 0.05). Glx showed significant positive correlations with NDVI, CCI, SPAD, ETo/RC (*p* ≤ 0.01), as well as Fv/Fm, PI_abs_, and PI_total_ (*p* ≤ 0.05). However, Glx was negatively correlated with DIo/Rc, TRo/RC, and RWC (*p* ≤ 0.01) and with ABS/RC (*p* ≤ 0.05). Alanine and proline showed no significant correlations with photosynthetic traits in UVE17 at the pod-filling stage under drought stress following selenium application.

Table 8 presents the correlation between photosynthetic efficiency and osmolyte accumulation in the vegetable-soybean cultivar UVE14 at the flowering stage under drought stress. Significant positive correlations were observed between TSS and CCI (*p* ≤ 0.05), as well as SPAD, DIo/RC (*p* ≤ 0.01), and TRo/RC (*p* ≤ 0.001). However, TSS was negatively correlated with Fv/Fm and ETo/RC (*p* ≤ 0.05). Starch, glucose, sucrose, and fructose levels were not significantly correlated with any of the measured physiological parameters. However, trehalose showed a significant negative correlation with ABS/RC (*p* ≤ 0.01), whereas raffinose was positively correlated with PRI (*p* ≤ 0.05). In addition, sucrose content was negatively correlated with Fv/Fm (*p* ≤ 0.05). Free amino acids (FAA) content showed significant positive correlations with ETo/RC (*p* ≤ 0.01) but was negatively correlated with NDVI and Chl *b* content (*p* ≤ 0.05). Arginine and glycine showed significant positive correlations with total chlorophyll content (*p* ≤ 0.05 and *p* ≤ 0.01, respectively). Asx had significant positive correlations with SPAD and Chl *b* (*p* ≤ 0.05), as well as with DIo/RC and TRo/RC (*p* ≤ 0.01). In contrast, Asx was negatively correlated with Fv/Fm and ETo/RC (*p* ≤ 0.05), and with PI_abs_ (*p* ≤ 0.01). Glx had positive correlations with DIo/RC and Chl *b* and negative correlations with ETo/RC (all *p* ≤ 0.01). Alanine and proline showed no significant correlations with the measured photosynthetic traits in UVE14 at the flowering stage under drought stress following selenium application.

Table 9 represents the correlation between photosynthetic parameters and osmolyte accumulation in the vegetable-soybean cultivar UVE14 at the pod-filling stage under drought stress is shown at. Total soluble sugars showed significant positive correlations with DIo/RC (*p* ≤ 0.01). Fructose, sucrose, and trehalose content were not significantly correlated with any of the measured physiological parameters. Glucose and raffinose content showed significant negative (*p* ≤ 0.05) and positive (*p* ≤ 0.01) correlations with NDVI. Free amino acid content showed a significant negative correlation with PI_total_ (*p* ≤ 0.05). Arginine was negatively correlated with CCI (*p* ≤ 0.01) and Chl *b* (*p* ≤ 0.05). Asx showed significant positive correlations with PRI and ETo/RC (*p* ≤ 0.001), as well as with PI_abs_, RWC, and Chl *a* (*p* ≤ 0.01). In contrast, Asx was negatively correlated with SPAD and Chl *b (p* ≤ 0.01). Glx showed a significant positive correlation with Chl *a* content (*p* ≤ 0.05).

## 3. Discussion

Overall, the results demonstrated that the response of vegetable-soybean to selenium under drought stress is highly dependent on both cultivar and water regime, highlighting the context-dependent nature of selenium-mediated stress tolerance. Selenium is closely related to amino acid metabolism in plants [35], and this relationship was evident in the present study, where soil-applied selenium enhanced free amino acid accumulation under drought stress at the flowering stage. Selenium is chemically similar to sulfur; consequently, it may influence the synthesis and accumulation of sulfur-containing amino acids, particularly cysteine and methionine, by competing with sulfur for uptake, assimilation, and incorporation into amino acid biosynthetic pathways [36]. Although these amino acids were not significantly affected in our results, this mechanism is included to provide a broader biochemical context for the observed responses of other amino acids. The accumulation of amino acids is a well-recognized adaptive response to abiotic stress, contributing to osmotic adjustment and cellular protection against desiccation [37,38]. Enhanced amino acid levels support essential physiological processes, thereby reducing drought-induced damage and improving stress resilience [20,39].

However, not all amino acids function solely as osmolytes during drought stress. Some amino acids, including asparagine, aspartate, and glutamate, perform broader metabolic functions, acting as key components in nitrogen storage and transport, and as substrates for the synthesis of other metabolites. Selenium-induced increases in aspartate and asparagine levels have previously been reported in potato tubers, where they support nitrogen storage and redistribution [35]. Similarly, glutamate, alanine, and aspartate showed significant accumulation in wheat leaves and roots under salt-stress, reflecting their involvement in both osmotic and metabolic adjustments [40,41]. Beyond their role in osmotic balance, these amino acids act as precursors for antioxidants, vitamins, and cofactors, thereby supporting cellular homeostasis and antioxidant defense mechanisms under stress conditions [42]. In this study, soil-applied selenium preferentially enhanced the accumulation of aspartate, asparagine, glutamine, and glutamate in the drought-susceptible UVE17 cultivar under drought conditions, suggesting cultivar-specific metabolic reprogramming in response to selenium. Aspartate and glutamate have been reported to contribute substantially to drought resilience by stabilizing proteins and cellular structures and by serving as precursors for other compatible solutes and chlorophyll biosynthesis [19,43]. In drought-tolerant cultivars, significant positive correlations were observed between Asx and Glx and chlorophyll *b* content. Moreover, Glx showed significant positive correlations with PI_abs_ and PI_total_ in drought-susceptible cultivar UVE17, supporting the role of amino acids in maintaining protein stability and cellular integrity. This may contribute to reduced chlorophyll degradation, thereby improving thylakoid function and photosynthetic performance. Conversely, reduced Glx levels at the pod-filling stage were associated with chlorophyll degradation and a decline in photochemical efficiency.

Amino acids, including proline, alanine, glycine, and asparagine, are frequently associated with abiotic stress tolerance [44]. Proline is often considered a dominant compatible solute that contributes to membrane stabilization and protection against desiccation under stressful conditions [28]. However, in the present study, drought stress did not enhance the accumulation of alanine, arginine, or proline, suggesting that the stress imposed did not cause severe cellular damage. This interpretation is consistent with reports indicating that lower proline accumulation may reflect reduced stress-induced damage rather than an impaired stress response [28]. Moreover, soil-applied selenium did not promote the accumulation of these amino acids. The lack of accumulation of these amino acids further suggests that the plants experienced limited injury. Comparable observations have been reported in field-grown potatoes, where proline levels remained unchanged under stress conditions following selenium application [28]. In contrast to our study, selenium-induced proline accumulation has been documented under more severe or acute stress conditions, such as salt stress, in wheat and onion plants [32,45], highlighting the strong dependence of amino acid responses on stress type and intensity. Indeed, amino acid concentration and composition are known to vary with stress severity, affected organ, and plant developmental stage [46].

Consistent with this developmental regulation, amino acid levels were significantly reduced in the later developmental stage (pod-filling) compared with the flowering stage when selenium was applied. This reduction may suggest a decreased demand for osmoprotective amino acids in leaves as stress intensity declined and the plant progressed through reproductive development. At this stage, amino acids are increasingly remobilized from the source tissues to developing sinks, particularly seeds, to support protein synthesis and nutrient accumulation. However, there is limited direct evidence linking selenium application to reduced leaf amino acid pools during pod-filling. Moreover, to our knowledge, no previous studies have reported reduced amino acid levels at the pod-filling stage following selenium application. However, Chilimba et al. [47] reported that selenium fertilization increased selenium bioavailability in edible crop products. We hypothesize that the reduction observed in the present study may be associated with the remobilization of amino acids to developing seeds. In addition to amino acids, soluble sugars, which also function as osmolytes, were investigated following selenium application. Given this shift in osmotic and metabolic regulation across developmental stages, the role of soluble sugars as alternative or complementary osmolytes under selenium application was further examined.

Soluble sugars, including glucose, sucrose, and fructose, serve dual functions in plants: as primary energy sources and contributors to osmotic adjustment and the maintenance of leaf water content under drought stress [15]. The accumulation of total soluble sugars may arise from improved photosynthetic carbon assimilation or from the mobilization of stored carbohydrates through starch degradation [28,48]. In the current study, TSS increased significantly under well-watered conditions in UVE17 following selenium soil drenching; however under drought stress, selenium application did not enhance total soluble sugar levels. This response is consistent with previous findings in selenium-treated soybean plants grown under optimal water availability, where increased sugar accumulation was linked to improved photosynthetic performance [49]. In contrast, selenium supplementation in drought-stressed tomato seedlings did not increase soluble sugar levels [50], suggesting that selenium-mediated regulation of sugar metabolism is strongly dependent on water status and species-specific physiological responses.

The observed increase in soluble sugars under well-watered conditions supports the notion that selenium can directly enhance photosynthetic efficiency, thereby increasing carbon availability for sugar synthesis. Notably, despite the absence of a significant increase in total soluble sugars under drought stress, selenium application markedly improved photosynthetic performance in both cultivars. This suggests that the lack of sugar accumulation under drought does not necessarily reflect impaired photosynthesis. However, future studies should investigate the activity of starch-hydrolyzing enzymes to further elucidate the mechanisms underlying enhanced photosynthesis without a corresponding increase in total soluble sugar levels under drought stress.

Raffinose emerged as a key soluble sugar in response to selenium application, and its accumulation was observed under well-watered conditions in cultivar UVE17. This increase largely accounted for the selenium-induced increase in total soluble sugars, while fructose and trehalose showed no significant changes at the flowering stage. Fructose, trehalose, and raffinose have been reported to contribute to drought tolerance through osmoprotection and the stabilization of cellular structures [51,52]. In addition, raffinose plays an important role in plant growth and development, particularly under non-stress conditions [53]. The preferential accumulation of raffinose under well-watered conditions suggests that selenium may promote carbohydrate partitioning towards raffinose synthesis to support metabolic stability and growth rather than stress defense. This response further supports the notion that selenium modulates carbohydrate metabolism in a cultivar- and condition-specific manner. Notably, reports describing raffinose accumulation in response to selenium application remain scarce, highlighting the novelty of the present findings and the need for further investigation into the role of selenium in regulating raffinose family oligosaccharide metabolism.

Two-way interaction effects further revealed that selenium-mediated regulation of soluble sugars was highly dependent on cultivar and water availability. Notably, sucrose content increased in cultivar UVE14 following selenium soil drenching, indicating enhanced carbon assimilation and translocation [54]. Du et al. [55] reported that increased sucrose accumulation in drought-stressed soybean plants was associated with enhanced photosynthetic efficiency. Glucose content responded to selenium only under water stress. Collectively, these findings indicate that selenium application in edamame affects soluble sugar metabolism in a cultivar- and soil water condition-specific manner.

Photosynthesis is the primary process influencing soluble sugar synthesis and plant growth [7]. The observed changes in sugar profiles were closely aligned with selenium-induced improvements in photosynthetic performance. Selenium application enhanced key chlorophyll fluorescence parameters, including PI_abs_, PI_total,_ and Fv/Fm, under well-watered conditions in both cultivars, indicating improved photochemical efficiency and photosystem stability. These findings corroborate previous reports showing that selenium soil drenching increased PI_abs_ and PI_total_ in UVE17 under water-limited conditions [3]. These responses highlight selenium’s capacity to sustain photosynthetic efficiency and physiological stability under drought stress.

In addition to improvements in chlorophyll fluorescence, selenium soil drenching enhanced chlorophyll pigment content and leaf greenness, as evidenced by increased NDVI and SPAD values in UVE17 under drought stress. Similarly, nano-selenium application enhanced SPAD values in drought-stressed soybean plants [6]. Moreover, selenium application increased chlorophyll *a*, chlorophyll *b*, and total chlorophyll contents in UVE14 at the pod-filling stage, indicating delayed chlorophyll degradation during later developmental stages. Significant positive correlations were observed between NDVI and SPAD values and free amino acid accumulation. This finding supports the role of amino acids, especially glutamate, in chlorophyll biosynthesis, thereby contributing to improved photosynthetic efficiency [21]. Similarly, selenium-induced maintenance of chlorophyll pigments has been reported in soybean plants under drought stress [6]. These findings indicate that selenium soil application mitigates chlorophyll degradation and leaf senescence during later developmental stages, which is important for maintaining photosynthetic activity under stress conditions.

## 4. Materials and Methods

### 4.1. Plant Material, Experimental Setup, and Treatments

The trials were conducted in the greenhouse of the University of the Free State, Bloemfontein campus (29°6′31.94″ S; 26°11′18.95″ E). Two vegetable-soybean cultivars provided by the Edamame Development Program (EDP), the drought-tolerant UVE14 and the drought-susceptible UVE17, were germinated under controlled conditions (25 °C day/18 °C night) in polystyrene floating seedling trays (128 cells) filled with Hygromix seedling substrate (Hygrotech (Pty) Ltd., Pretoria, South Africa). Seedlings were watered daily until the emergence of the first trifoliate leaf, corresponding to vegetative stage 1 (V1). An illustration of the vegetative and reproductive growth stages of vegetable-soybean is provided in Supplementary Figure S1.

At the V1 stage, seedlings were transferred into potting bags (10 L capacity, 350 mm length, 150 mm width) containing 10 kg of sandy loamy soil and maintained at 100% soil water-holding capacity (WHC). The soil had a pH of 8.64, a redox potential of 217 mV, and an electrical conductivity (EC) of 41 µS cm^−1^. At the V3 stage, 200 mL of a 50 mg/L sodium selenate (Na_2_SeO_4_) solution (Sigma-Aldrich, Saint Louis, MO, USA) was applied to the soil while maintaining 100% soil WHC. Control plants received the same amount of water without selenium. The optimal selenium concentration, applied volume, and soil application method were established in previous studies [33,34,56]. At the V4 stage, drought stress was induced by reducing soil moisture to 30% soil WHC, whereas well-watered control plants were maintained at 100% soil WHC. A Hydrosense II meter (CS659; Campbell Scientific, Stellenbosch, South Africa) was used to monitor soil moisture and maintain the respective watering regimes throughout the experiment. This resulted in four treatment combinations: (i) no selenium + 100% soil WHC (positive control); (ii) no selenium + 30% soil WHC (negative control); (iii) selenium +100% soil WHC (selenium treatment control); and (iv) selenium + 30% soil WHC (experimental treatment). To prevent nutrient deficiency, plants were fertilized every two weeks with 200 mL of Hygrotech nutrient solution and NPKC (Wonder plant starter granular fertilizer, Agro-Serve (Pty) Ltd., Islando, South Africa), which supplied essential macro- and micronutrients throughout the experimental period. The experiment was arranged in a randomized complete block design (RCBD) with three replicates and four pots per replicate.

Sampling for both non-destructive and destructive measurements was performed at the flowering stage (R2) and pod-filling stage (R4) between 9:00 a.m. and 12:00 p.m. on young, fully expanded trifoliate leaves. For destructive analyses, the leaves were harvested and immediately frozen in liquid nitrogen. In the laboratory, leaves from each replicate were ground to a fine powder in liquid nitrogen and stored at −20 °C until analysis.

### 4.2. Photosynthesis Parameters

#### 4.2.1. Chlorophyll *a* Fluorescence

Photosynthesis is a primary process influencing plant growth and grain yield [7]. Plant health can be monitored in vivo using non-destructive techniques. In this study, a leaf spectrometer (CID Bio Science, International Light Technologies Inc., Camas, WA, USA) was used to measure the photochemical reflectance index (PRI), normalized difference vegetative index (NDVI), Soil–Plant Analysis Development (SPAD) for the relative chlorophyll index, and chlorophyll content index (CCI). Chlorophyll *a* fluorescence, measured using a Pocket photosynthesis efficiency analyzer (PEA), (Hansatech Instruments Ltd., Amesbury, MA, USA), was used to assess the structure and function of photosystem II (PSII) under stress conditions, providing insights into the status of reaction centers and antenna complexes [57,58]. Sample leaves were dark-adapted for 30 min using lightweight leaf clips with closed shutter plates (one clip per representative leaf per plant) to exclude light. The Pocket PEA sensor was then attached to the clip, followed by opening of the shutter and exposure to a saturating light pulse (3500 μmol m^−2^s^−1^). Photochemical efficiency parameters were measured using PEA Plus software (version 1.10, Hansatech Instruments Ltd., Amesbury, MA, USA). The following parameters were evaluated according to Kalaji et al. [57]: maximum photosystem II (PSII) quantum yield (ratio of variable to maximum fluorescence (Fv/Fm), total performance index (PI_total_), performance index absorbance (PI_abs_), energy absorbed per reaction center (RC) (ABS/RC), energy dissipated as heat per RC (DIo/RC), trapped energy per RC (TRo/RC), and the flux of electrons transferred from quinone (QA) to plastoquinone (PQ) per active PSII RC (ETo/RC).

#### 4.2.2. Chlorophyll Content

Chlorophyll plays a key role in photosynthesis, and its content is known to change in response to various stresses [59]. Chlorophyll content was determined according to the method described by Su et al. [60]. Frozen leaf samples (0.1 g) were extracted with 5 mL of ice-cold 80% (*v*/*v*) acetone (Sigma-Aldrich, Saint Louis, MO, USA) using a pestle and mortar on ice. The extract was centrifuged at 11,000× *g* for 10 min. The absorbance of the supernatant was measured at 663 and 645 nm. The following equations were used for the calculations:

Chlorophyll *a* = [(12.72 × OD_663nm_) − (2.59 × OD_645nm_)]

Chlorophyll *b* = [(22.9 × OD_645nm_) − (4.68 × OD_663nm_)]

Total chlorophyll = 20.2 OD_645nm_ + 8.02 OD_663nm_

### 4.3. Relative Water Content

Relative water content (RWC) is a measure of the plant water status. It reflects the metabolic activity in plant tissues and is widely used as an indicator of dehydration tolerance [7]. Relative water content was determined according to the method described by Gonzáles and Gonzáles-Vilar [61]. At the R2 and R4 growth stages, young, fully developed leaves were collected and placed in airtight Falcon tubes (50 mL) to prevent moisture loss between harvesting and RWC analysis in the laboratory. In the laboratory, leaf fresh weight (FW) was recorded immediately. After weighing, 10 mL of double-distilled water was added to each Falcon tube, and the samples were incubated at 4 °C for 24 h. The leaves were gently blotted dry with paper towels, and the turgid weight (TW) was recorded. The leaves were placed in paper bags and dried at 72 °C for 72 h. The dry weight (DW) was recorded, and RWC was calculated as follows:

RWC (%) = (FW − DW) (TW − DW) × 100

### 4.4. Free Amino Acids Quantification

Free amino acids (FAAs) act as nitrogen buffers under drought conditions, helping to stabilize protein metabolism and sustain physiological processes, thereby improving drought tolerance [62]. Quantification of free amino acids in the supernatant was performed using the ninhydrin reaction [63]. Total free amino acids were extracted from frozen leaf samples (0.25 mg) with 5 mL of double-distilled water in a water bath at 95 °C for 45 min. The extracts were centrifuged at 10,000× *g* for 10 min at 4 °C. The supernatant (400 μL) was mixed with 800 μL of ninhydrin solution [0.8 g of ninhydrin (Sigma-Aldrich, Saint Louis, MO, USA) dissolved in 30 mL of 2-methoxyethanol (Sigma-Aldrich, Saint Louis, MO, USA) plus 10 mL of acetate (Sigma-Aldrich, Saint Louis, MO, USA) buffer 4 M, pH 5.5], and heated at 95 °C for 15 min. After cooling, 1 mL 50% (*v*/*v*) ethanol (Sigma-Aldrich, Saint Louis, MO, USA) was added to the reaction mixture, which was then incubated at room temperature for 10 min. Absorbance was then measured at 570 nm. Total amino acid content was quantified using a glycine (Merk, Darmstadt, Germany) standard curve.

### 4.5. Soluble Carbohydrates Quantification

#### 4.5.1. Total Soluble Sugars

Carbohydrate metabolism is affected by drought and is considered a critical physiological trait influencing plant growth and yield in vegetable-soybean [13,16]. Total soluble sugars (TSS) were determined according to the method described by Irigoyen et al. [64] Frozen leaf powder (0.1 g) was homogenized in 96% (*v*/*v*) ethanol (Sigma-Aldrich, Saint Louis, MO, USA) and incubated at 80 °C for 10 min. The extraction mixture was centrifuged at 4000× *g* for 10 min at 4 °C, and the supernatant containing soluble sugars was collected. An aliquot (50 µL) of ethanoic extract was added to 1450 µL of anthrone reagent 1.5 mg anthrone (Sigma-Aldrich, Saint Louis, MO, USA) dissolved in 100 mL of 72% (*v*/*v*) sulfuric acid (Merk, Darmstadt, Germany). The mixture was vortexed vigorously and incubated at 80 °C for 15 min. Absorbance was measured at 625 nm using a spectrophotometer (Cary 100 Bio, Varian, Australia). The TSS concentration was determined using a glucose (Merk, Darmstadt, Germany) standard curve.

#### 4.5.2. Glucose, Trehalose, Raffinose, Fructose, and Sucrose

Soluble sugars such as glucose, trehalose, and sucrose are known to improve drought tolerance in plants by acting as osmolytes, preventing oxidative stress, and stabilizing the plasma membranes [13]. Soluble sugars were extracted twice from 0.1 g of ground leaf tissue with 2 mL of 80% (*v*/*v*) ethanol (Sigma-Aldrich, Saint Louis, MO, USA) at 80 °C. Each extraction step was performed for 15 min, followed by centrifugation at 10,000× *g* for 4 min. Activated charcoal (80 mg) (Merck, Darmstadt, Germany) was added to the supernatant to remove other non-sugar leaf metabolites. The mixture was mixed, incubated at room temperature for 5 min, and then centrifuged at 3000× *g* for 15 min.

Glucose, trehalose, sucrose, and raffinose contents in the supernatant were quantified according to a modified method described by Hlahla et al. [13]. The sucrose assay was initiated by adding invertase (30.9 U mg^−1^; BDH Biochemicals, London, UK) to tubes containing 0.05 mL of soluble sugar extract and 0.150 mL of 50 mM sodium phosphate (Sigma-Aldrich, Saint Louis, MO, USA) buffer at pH 6.5. The mixture was incubated at 30 °C for 15 min to catalyze the hydrolysis of sucrose into D-glucose and D-fructose. Glucose content was quantified using GOPOD (Megazyme, Wicklow, Ireland) after incubation of the samples with amylase (Megazyme, Wicklow, Ireland) at 80 °C for 30 min, followed by incubation with β-glucosidase at 50 °C for further 30 min (Megazyme, Wicklow, Ireland). Fructose, trehalose, and raffinose contents were quantified using reagent kits obtained from Megazyme (Wicklow, Ireland).

### 4.6. Statistical Analysis

Statistical analysis of photosynthetic and osmolyte parameters were performed using Statistica version 7 (TIBCO Software Inc., Palo Alto, CA, USA). The individual and combined effects of selenium soil application, water level, and cultivar were evaluated using multifactorial analysis of variance (MANOVA). Fisher’s least significant difference (LSD) post hoc test was used to determine the difference among treatment means at *p* ≤ 0.05. Relationships between photosynthetic parameters and osmolyte levels were assessed using correlation matrices generated with the two-variable list matrix option, which provided regression coefficients and *p*-values at the 95% confidence level.

## 5. Conclusions

This study demonstrates that selenium soil application modulates osmolyte metabolism and photosynthetic performance in vegetable soybean, with effects strongly dependent on cultivar, water availability, and developmental stage. Under drought stress, selenium enhanced drought tolerance primarily by modulating amino acid metabolism at flowering, with increased accumulation of Asx and Glx accompanying improvements in chlorophyll *b* content, photosynthetic indices, and PSII photochemical efficiency, indicating coordinated regulation between nitrogen metabolism and photosynthetic function. While both cultivars responded positively to selenium under drought conditions, the magnitude and consistency of selenium-induced improvements in photosynthetic efficiency (Fv/Fm, PI_abs_, PI_total_) and pigment-related traits (CCI, SPAD, NDVI) were more evident in UVE17, particularly at the flowering stage, suggesting greater physiological responsiveness to selenium in this cultivar under water-limited conditions. In contrast, the effects of selenium on soluble sugars were more pronounced under well-watered conditions and varied between cultivars, indicating that carbon-based adjustment mechanisms in edamame are more prominent when water is not limited. The limited accumulation of common stress metabolites such as proline further suggests that selenium mitigates drought-induced cellular disruption rather than triggering severe stress responses. At the pod-filling stage, reduced leaf amino acid levels following selenium application likely reflected enhanced nitrogen remobilization to developing seeds. Overall, these findings highlight selenium as a context-dependent modulator of drought resilience and emphasize the importance of cultivar- and growth-stage-specific optimization of selenium application strategies.

## Figures and Tables

**Table 1:** Mean values of amino acids showing significant cultivar × water level × selenium (C × W × Se) interactions in the vegetable-soybean cultivars UVE14 and UVE17 at the flowering and pod-filling stages.

	Asx (pmol^−1^ ul^−1^ g^−1^)	Glx (pmol^−1^ ul^−1^ g^−1^)
**Flowering**		
UVE14 100% soil WHC − Se	0.48 ^ab^	1.10 ^a^
UVE14 100% soil WHC + Se	0.95 ^cd^	1.61 ^cd^
UVE17 100% soil WHC − Se	0.77 ^bc^	1.22 ^a^
UVE17 100% soil WHC + Se	0.74 ^abc^	1.30 ^bcd^
UVE14 30% soil WHC − Se	1.14 ^d^	1.63 ^d^
UVE14 30% soil WHC + Se	0.86 ^cd^	1.23 ^abc^
UVE17 30% soil WHC − Se	0.45 ^a^	0.64 ^a^
UVE17 30% soil WHC + Se	0.87 ^cd^	1.14 ^bc^
**Pod-filling**		
UVE14 100% soil WHC − Se	0.42 ^b^	0.35 ^b^
UVE14 100% soil WHC + Se	0.019 ^a^	0.02 ^a^
UVE17 100% soil WHC − Se	0.039 ^a^	0.04 ^a^
UVE17 100% soil WHC + Se	0.033 ^a^	0.05 ^a^
UVE14 30% soil WHC − Se	0.007 ^a^	0.15 ^a^
UVE14 30% soil WHC + Se	0.023 ^a^	0.04 ^a^
UVE17 30% soil WHC − Se	0.001 ^a^	0.02 ^a^
UVE17 30% soil WHC + Se	0.015 ^a^	0.04 ^a^

Values represent means ± SE (n = 3). Fisher’s LSD post hoc test was used to determine the significance of differences among treatment means (*p* < 0.05). Treatments with different letters within columns are not significantly different. UVE14: drought-tolerant cultivar; UVE17: drought-susceptible cultivar. Plants were grown under 100% and 30% soil water-holding capacity (WHC). Selenium treatments were designated as +Se (selenium application) and −Se (no selenium application). Asx = asparagine + aspartate, Glx = glutamate + glutamine.

**Table 2:** Amino acid concentrations with a significant effect of water treatment at the flowering and pod-filling stages.

	Flowering			Pod-Filling		
Water Level	Arg (pmol^−1^ ul^−1^ g^−1^)	Ala (pmol^−1^ ul^−1^ g^−1^)	Pro (pmol^−1^ ul^−1^ g^−1^)	Arg (pmol^−1^ ul^−1^ g^−1^)	Ala (pmol^−1^ ul^−1^ g^−1^)	Pro (pmol^−1^ ul^−1^ g^−1^)
100% soil WHC	0.75 ^b^	0.46 ^b^	0.51 ^b^	0.002 ^a^	0.04 ^a^	0.04 ^a^
30% soil WHC	0.44 ^a^	0.38 ^a^	0.29 ^a^	0.05 ^a^	0.005 ^a^	0.01 ^a^

Values represent means ± SE (n = 3). Fisher’s LSD post hoc test was used to determine significant differences between treatment means (*p* < 0.05). Treatments with different letters (within a column) are significantly different. Treatments sharing one or more letters are not statistically different. UVE14: drought-tolerant cultivar; UVE17: drought-susceptible cultivar. Plants were grown under 100% and 30% soil water-holding capacity (WHC). Selenium treatments were designated as +Se (selenium application) and −Se (no selenium application). Arg = arginine, Ala = alanine, Pro = proline.

**Table 3:** Mean values of photosynthetic efficiency parameters showing significant Cultivar × Water × Selenium interactions in two vegetable-soybean cultivars at the flowering and pod filling stages.

Treatment	CCI	NDVI	SPAD	Fv/Fm (a.u)	ABS/RC (a.u)	PI_abs_ (a.u)	PI_total_ (a.u)	Chl *a* (mg/g FW)
**Flowering**								
UVE14 100% soil WHC − Se	12.05 ^cd^	0.67 ^a^	28.74 ^c^	0.77 ^a^	2.09 ^e^	1.10 ^a^	0.82 ^a^	1.08 ^de^
UVE14 100% soil WHC + Se	13.93 ^e^	0.67 ^a^	31.09 ^e^	0.78 ^a^	1.80 ^d^	1.91 ^b^	1.50 ^b^	1.19 ^e^
UVE17 100% soil WHC − Se	13.61 ^d^	0.69 ^b^	30.05 ^d^	0.77 ^a^	1.87 ^d^	1.10 ^a^	0.87 ^a^	0.70 ^a^
UVE17 100% soil WHC + Se	12.23 ^cd^	0.67 ^a^	28.95 ^c^	0.79 ^ab^	1.62 ^c^	2.72 ^c^	1.52 ^b^	0.91 ^bc^
UVE14 30% soil WHC − Se	12.54 ^d^	0.67 ^a^	28.89 ^c^	0.81 ^bcd^	1.50 ^b^	1.98 ^b^	1.65 ^bc^	1.02 ^cd^
UVE14 30% soil WHC + Se	10.76 ^ab^	0.67 ^a^	27.17 ^b^	0.81 ^cd^	1.22 ^a^	3.22 ^d^	1.90 ^c^	1.02 ^cd^
UVE17 30% soil WHC − Se	10.05 ^a^	0.67 ^a^	25.82 ^a^	0.80 ^bc^	1.17 ^a^	3.86 ^e^	2.79 ^d^	0.89 ^b^
UVE17 30% soil WHC + Se	11.64 ^bc^	0.69 ^b^	28.82 ^c^	0.83 ^d^	1.11 ^a^	5.95 ^f^	4.44 ^e^	0.85 ^b^
**Pod-filling**								
UVE14 100% soil WHC − Se	12.05 ^bc^	0.673 ^bc^	28.74 ^c^	0.74 ^a^	2.06 ^d^	0.45 ^a^	1.37 ^ab^	0.74 ^c^
UVE14 100% soil WHC + Se	13.93 ^d^	0.67 ^ab^	31.09 ^e^	0.77 ^b^	1.61 ^c^	1.49 ^b^	1.59 ^b^	0.99 ^d^
UVE17 100% soil WHC − Se	13.61 ^d^	0.686 ^d^	30.05 ^d^	0.74 ^a^	2.02 ^d^	0.54 ^a^	1.07 ^a^	0.53 ^a^
UVE17 100% soil WHC + Se	12.23 ^c^	0.67 ^bc^	28.95 ^c^	0.77 ^b^	1.60 ^c^	1.73 ^b^	1.68 ^b^	0.66 ^b^
UVE14 30% soil WHC − Se	11.89 ^bc^	0.67 ^bc^	28.31 ^c^	0.77 ^b^	1.39 ^b^	2.56 ^c^	2.17 ^c^	1.11 ^e^
UVE14 30% soil WHC + Se	10.48 ^a^	0.66 ^a^	26.64 ^a^	0.78 ^b^	1.34 ^b^	3.12 ^d^	2.23 ^c^	1.25 ^f^
UVE17 30% soil WHC − Se	10.05 ^a^	0.68 ^c^	25.82 ^a^	0.81 ^c^	1.15 ^a^	5.66 ^f^	3.11 ^e^	1.14 ^e^
UVE17 30% soil WHC + Se	11.32 ^b^	0.69 ^e^	27.67 ^b^	0.81 ^c^	1.22 ^a^	3.66 ^e^	2.69 ^d^	1.08 ^e^

Values represent means ± SE (n = 3). Fisher’s LSD post hoc test was used to determine the significance of the differences between averages (*p* < 0.05). Treatments with different letters (within a column) are significantly different. Treatments sharing one or more letters are not statistically different. Plants were grown under 100% and 30% soil water-holding capacity (WHC) representing well-watered and drought conditions. Selenium treatments were designated as +Se (selenium application) and −Se (no selenium application). UVE14 = drought-tolerant cultivar; UVE17 = drought-susceptible cultivar. NDVI = Normalized difference vegetative index, CCI = Chlorophyll content Index, SPAD = Structure intensive pigment index, Fv/Fm = maximum photosystem II (PSII) quantum yield ratio of variable to maximum fluorescence, ABS/RC = energy absorbed per reaction center, PI_abs_ = total performance index, PI_total_ = total performance index, Chl *a* = chlorophyll *a*. Au= arbitrary units.

**Table 4:** Photosynthetic parameters showing significant Water × Selenium interactions in vegetable-soybean at flowering and pod-filling stages.

		Flowering				Pod-Filling			
Water	Selenium	PRI (a.u)	DIo/RC (a.u)	TRo/RC (a.u)	ETo/RC (a.u)	PRI (a.u)	DIo/RC (a.u)	TRo/RC (a.u)	ETo/RC (a.u)
100% soil WHC	−Se	0.0030 ^a^	0.60 ^d^	1.59 ^d^	0.39 ^a^	0.02 ^b^	0.45 ^d^	1.52 ^c^	0.55 ^c^
100% soil WHC	+Se	0.0039 ^a^	0.37 ^c^	1.22 ^c^	0.49 ^bc^	0.02 ^b^	0.35 ^c^	1.24 ^b^	0.50 ^b^
30% soil WHC	−Se	0.022 ^c^	0.30 ^b^	1.04 ^b^	0.47 ^b^	0.014 ^a^	0.24 ^b^	1.02 ^a^	0.44 ^a^
30% soil WHC	+Se	0.016 ^b^	0.26 ^a^	0.98 ^a^	0.53 ^c^	0.017 ^a^	0.20 ^a^	1.03 ^a^	0.53 ^c^

Values represent means ± SE (n = 3). Fisher’s LSD post hoc test was used to determine the significance of differences between the treatment means (*p* < 0.05). Treatments with different letters (within a column) are significantly different. Treatments sharing one or more letters. are not statistically different. UVE14: drought-tolerant cultivar; UVE17: drought-susceptible cultivar. Plants were grown under 100% and 30% soil water-holding capacity (WHC), representing well-watered and drought conditions, respectively. Selenium treatments were designated as and selenium application as +Se (selenium application) and −Se (no selenium application). PRI = Photochemical reflectance index, ABS/RC = energy absorbed per reaction center, DIo/RC = energy dissipated as heat per reaction center. TRo/RC = trapped energy per reaction center, ETo/RC = the flux of electrons transferred from quinone. (QA) to plastoquinone (PQ) per active PSII per reaction center.

**Table 5:** Chlorophyll pigments showing significant Cultivar × Selenium interactions in vegetable-soybean at flowering and pod-filling stages.

		Flowering		Pod-Filling	
Cultivar	Selenium	Chl *b* (mg/g FW)	Tot Chl (mg/g FW)	Chl *b* (mg/g FW)	Tot Chl (mg/g FW)
UVE14	−Se	0.77 ^c^	1.17 ^a^	1.759 ^c^	1.632 ^b^
UVE14	+Se	0.55 ^b^	1.29 ^b^	0.500 ^a^	1.759 ^b^
UVE17	−Se	0.38 ^a^	1.30 ^b^	0.377 ^a^	1.300 ^a^
UVE17	+Se	0.53 ^b^	1.68 ^b^	0.529 ^b^	1.677 ^b^

Values represent means ± SE (n = 3). Fisher’s LSD post hoc test was used to determine significant differences between treatment means (*p* < 0.05). Treatments with different letters (within a column) are significantly different. Treatments sharing one or more letters are not statistically different. UVE14: drought-tolerant cultivar; UVE17: drought-susceptible cultivar. Plants were grown under 100% and 30% soil water-holding capacity (WHC), representing well-watered and drought conditions, respectively. Selenium treatments were designated as +Se (selenium application) and −Se (no selenium application). Chl *b* = chlorophyll *b*, Tot Chl = total chlorophyll content.

**Table 6:** The correlation between photosynthetic efficiency and osmolyte accumulation of vegetable-soybean cultivar UVE17 at flowering stage under drought stress.

	TSS	Glucose	Sucrose	Fructose	Trehalose	Raffinose	FAA	Arg	Gly	Asx	Glx	Ala	Pro
PRI	0.28	0.16	−0.54	0.63	0.29	0.26	−0.82 *	−0.39	−0.40	−0.73	−0.64	−0.48	−0.42
NDVI	−0.36	0.00	0.51	−0.75	−0.38	−0.21	0.89 **	0.45	0.45	0.21	0.35	0.50	0.44
CCI	−0.90 **	0.34	0.83 *	−0.90 **	−0.31	0.13	0.89 **	0.75	0.69	0.51	0.36	0.79	0.77
SPAD	−0.83 *	0.39	0.74	−0.87 *	−0.39	0.18	0.96 **	0.66	0.59	0.59	0.40	0.72	0.68
Fv/Fm	0.77	−0.70	−0.55	0.57	−0.03	−0.26	−0.64	−0.11	−0.01	−0.58	−0.47	−0.14	−0.12
ABS/RC	−0.28	−0.24	0.47	−0.47	−0.49	0.01	0.65	0.63	0.63	0.07	0.05	0.74	0.69
DIo/RC	−0.36	−0.02	0.47	−0.60	−0.55	0.05	0.86 *	0.55	0.53	−0.24	−0.39	0.65	0.59
TRo/RC	−0.31	0.14	0.14	0.12	0.45	0.09	−0.50	0.08	0.05	0.44	0.40	−0.01	0.07
ETo/RC	−0.28	0.00	0.10	−0.34	−0.53	0.21	0.11	0.79	0.80	0.24	0.02	0.73	0.76
PI_abs_	0.62	−0.06	−0.72	0.91 **	0.37	0.16	−0.93 **	−0.70	−0.68	0.39	0.19	−0.73	−0.69
PI_total_	0.54	0.39	−0.77	0.68	0.10	0.33	−0.42	−0.89 *	−0.91 **	−0.46	−0.39	−0.89 *	−0.90 **
RWC	−0.22	0.86 *	−0.23	0.15	0.05	0.59	0.02	−0.47	−0.57	0.40	0.14	−0.47	−0.47
Chl *a*	0.49	−0.12	−0.33	0.70	0.67	−0.14	−0.55	−0.91 **	−0.90 **	0.55	0.37	−0.86 *	−0.86 *
Chl *b*	0.16	−0.11	0.01	−0.27	−0.49	−0.09	0.62	0.06	0.08	−0.61	−0.69	0.15	0.07
Tot Chl	−0.45	−0.28	0.78	−0.79	−0.05	−0.49	0.75	0.55	0.57	−0.70	−0.81 *	0.59	0.55

* *p* ≤ 0.05, ** *p* ≤ 0.01. TSS = total soluble sugars, FA = Free amino acids, Asx = Asparagine + Aspartate, Ala = Alanine, Arg = Arginine, Gly = Glycine, Glx = Glutamine + Glutamate, Pro = Proline, PRI = photochemical reflectance index, chlorophyll index, NDVI = normalized difference vegetative index, SPAD = structure intensive pigment index, CCI = Chlorophyll content Index, SPAD = structure intensive pigment index, Fv/Fm = maximum photosystem II (PSII) quantum yield ratio of variable to maximum fluorescence, ABS/RC = energy absorbed per reaction center, DI0/RC = energy dissipated as heat per reaction center. TRo/RC = trapped energy per reaction center, ETo/RC = the flux of electrons transferred from quinone (QA) to plastoquinone (PQ) per active PSII per reaction center, PI_abs_ = performance index absorbance, PI_total_ = total performance index, RWC = relative water content, Chl *a* = chlorophyll *a*, Chl *b* = chlorophyll *b*, Tot Chl = total chlorophyll content.

**Table 7:** The correlation between photosynthetic efficiency and osmolyte accumulation of vegetable-soybean cultivar UVE17 at pod-filling stages under drought stress.

	TSS	Glucose	Sucrose	Fructose	Trehalose	Raffinose	FAA	Arg	Gly	Asx	Glx	Ala	Pro
PRI	−0.73	0.89 *	0.29	−0.77	0.10	0.14	0.61	−0.43	−0.24	−0.67	−0.54	0.15	0.13
NDVI	0.70	−0.69	−0.37	0.90 **	−0.11	0.16	−0.77	0.50	0.21	0.7	0.92 **	−0.33	−0.32
CCI	−0.83 *	0.61	0.17	−0.15	−0.35	0.70	0.64	0.31	0.28	0.68	0.95 **	0.54	−0.11
SPAD	−0.43	−0.20	0.39	−0.25	−0.30	−0.05	0.74	0.34	0.39	0.79	0.94 **	0.70	0.01
Fv/Fm	0.27	−0.52	−0.15	0.86 *	−0.13	0.47	−0.51	0.83 *	0.59	0.77	0.85 *	0.11	−0.25
ABS/RC	−0.65	0.73	0.49	−0.92 **	0.23	−0.10	0.63	−0.45	−0.21	−0.45	−0.86 *	0.16	0.15
DIo/RC	−0.63	0.80	0.33	−0.86 *	0.14	−0.06	0.62	−0.55	−0.32	−0.93 **	−0.94 **	0.10	0.17
TRo/RC	−0.65	0.78	0.41	−0.89 **	0.16	−0.06	0.64	−0.49	−0.27	−0.83 *	−0.95 **	0.13	0.13
ETo/RC	0.55	−0.63	−0.37	0.94 **	−0.09	0.26	−0.72	0.60	0.36	0.72	0.96 **	−0.13	−0.17
PI_abs_	0.57	−0.75	−0.46	0.87 *	−0.16	0.02	−0.56	0.44	0.44	0.68	0.9 *	0.02	0.06
PI_total_	0.62	−0.66	−0.55	0.95 **	−0.30	0.18	−0.61	0.44	0.18	0.54	0.9 *	−0.17	−0.18
RWC	0.01	0.48	0.19	−0.23	0.65	0.08	−0.49	−0.15	−0.73	−0.93 **	−0.48	−0.01	−0.01
Chl *a*	0.68	−0.81	−0.38	0.85 *	−0.10	−0.03	−0.66	0.44	0.26	−0.31	−0.65	−0.13	−0.07
Chl *b*	0.63	−0.96 **	−0.14	0.55	−0.05	−0.35	−0.39	0.38	0.29	0.65	0.53	0.05	0.02
Tot Chl	0.76	−0.82 *	−0.48	0.84 *	−0.22	−0.08	−0.62	0.33	0.11	0.49	0.71	−0.22	−0.13

* *p* ≤ 0.05, ** *p* ≤ 0.01. TSS = total soluble sugars, FA = Free amino acids, Asx = Asparagine + Aspartate, Ala = Alanine, Arg = Arginine, Gly = Glycine, Glx = Glutamine + Glutamate, Pro = Proline, PRI = photochemical reflectance index, chlorophyll index, NDVI = normalized difference vegetative index, SPAD = structure intensive pigment index, CCI = chlorophyll content index, SPAD = structure intensive pigment index, Fv/Fm = maximum photosystem II (PSII) quantum yield ratio of variable to maximum fluorescence, ABS/RC = energy absorbed per reaction center, DI0/RC = energy dissipated as heat per reaction center. TRo/RC = trapped energy per reaction center, ETo/RC = the flux of electrons transferred from quinone (QA) to plastoquinone (PQ) per active PSII per reaction center, PI_abs_ = performance index absorbance, PI_total_ = total performance index, RWC = relative water content, Chl *a* = chlorophyll *a*, Chl *b* = chlorophyll *b*, Tot Chl = total chlorophyll content.

**Table 8:** The correlation between photosynthetic efficiency and osmolyte accumulation of vegetable-soybean cultivar UVE14 at the flowering stage under drought stress.

	TSS	Glucose	Sucrose	Fructose	Trehalose	Raffinose	FAA	Arg	Gly	Asx	Glx	Ala	Pro
PRI	0.41	0.22	−0.19	−0.52	0.22	0.82 *	0.16	−0.46	−0.49	0.34	−0.06	−0.36	−0.47
NDVI	0.40	−0.77	0.57	0.03	−0.75	−0.63	−0.83 *	0.48	0.57	0.51	0.77	0.70	0.77
CCI	0.87 *	−0.26	−0.02	−0.39	−0.10	0.20	−0.46	0.15	0.27	0.79	0.7	0.43	0.37
SPAD	0.91 **	−0.47	0.25	−0.66	−0.31	0.31	−0.61	0.04	0.07	0.8 *	0.68	0.36	0.28
Fv/Fm	−0.87 *	0.39	−0.23	0.64	0.31	−0.43	0.52	0.08	0.05	−0.81 *	−0.6	−0.23	−0.15
ABS/RC	0.10	−0.55	0.70	0.10	−0.83 *	0.14	−0.42	0.16	0.14	0.4	0.49	0.24	0.26
DIo/RC	0.90 **	−0.58	0.29	−0.39	−0.47	−0.06	−0.76	0.23	0.35	0.89 **	0.87 **	0.58	0.55
TRo/RC	0.98 ***	−0.46	0.18	−0.62	−0.34	0.12	−0.68	−0.04	0.04	0.92 **	0.69	0.32	0.27
ETo/RC	−0.83 *	0.77	−0.54	0.43	0.66	0.04	0.86 *	−0.34	−0.39	−0.82 *	−0.92 **	−0.67	−0.64
PI_abs_	−0.75	0.39	−0.31	0.41	0.52	−0.29	0.52	0.21	0.14	−0.89 **	−0.56	−0.08	−0.05
PI_total_	−0.52	0.05	0.12	0.62	−0.28	−0.46	0.12	−0.05	0.00	−0.2	−0.22	−0.19	−0.07
RWC	0.19	0.15	−0.25	−0.70	0.48	0.25	0.08	−0.28	−0.40	−0.19	−0.32	−0.23	−0.32
Chl *a*	0.35	0.12	−0.45	−0.26	0.28	−0.76	−0.22	−0.39	−0.26	0.22	−0.19	−0.22	−0.16
Chl *b*	0.75	−0.68	0.42	−0.17	−0.61	−0.24	−0.81 *	0.41	0.53	0.81 *	0.93 **	0.71	0.72
Tot Chl	−0.20	−0.21	0.21	0.75	−0.26	−0.28	−0.09	0.82 *	0.92 **	−0.03	0.54	0.72	0.78

* *p* ≤ 0.05, ** *p* ≤ 0.01, *** *p* ≤ 0.001. TSS = total soluble sugars, FAA = Free amino acids, Asx = Arginine + Aspartate, Ala = Alanine, Glx = Glutamate + Glutamine, Arg = Arginine, Gly = Glycine, Pro = Proline, PRI = photochemical reflectance index, NDVI = normalized difference vegetative index, SPAD = structure intensive pigment index, CCI = chlorophyll content index, Fv/Fm = maximum photosystem II (PSII) quantum yield ratio of variable to maximum fluorescence, ABS/RC = energy absorbed per reaction center, DIo/RC = energy dissipated as heat per reaction center. TRo/RC = trapped energy per reaction center, ETo/RC = the flux of electrons transferred from quinone (QA) to plastoquinone (PQ) per active PSII per reaction center, PI_abs_ = performance index absorbance, PI_total_ =total performance index, RWC = relative water content, Chl *a* = chlorophyll *a*, Chl *b* = chlorophyll *b*, Tot Chl = total chlorophyll content.

**Table 9:** The correlation between photosynthetic efficiency and osmolyte accumulation of vegetable-soybean cultivar UVE14 at the pod-filling stage under drought stress.

	TSS	Glucose	Sucrose	Fructose	Trehalose	Raffinose	FAA	Arg	Gly	Asx	Glx	Ala	Pro
PRI	−0.29	0.03	−0.09	−0.31	0.45	−0.09	−0.41	0.75	0.89 *	0.98 ***	0.61	0.73	0.66
NDVI	−0.38	−0.85 *	0.13	0.00	0.46	0.94 **	0.23	−0.48	−0.17	−0.23	0.59	−0.23	−0.14
CCI	0.56	−0.11	0.14	0.61	−0.11	0.20	0.44	−0.92 **	−0.61	−0.78	−0.54	−0.44	−0.44
SPAD	0.37	−0.04	−0.15	0.57	−0.34	0.05	0.60	−0.69	−0.72	−0.93 **	−0.68	−0.50	−0.43
Fv/Fm	−0.73	0.27	−0.52	−0.31	−0.54	−0.17	0.25	0.65	−0.03	0.02	−0.09	0.06	0.19
ABS/RC	0.54	0.23	−0.07	0.68	−0.35	−0.07	0.55	−0.71	−0.51	−0.69	−0.79	−0.26	−0.25
DIo/RC	0.94 **	0.14	0.30	0.38	0.10	−0.40	−0.19	−0.45	−0.19	−0.27	−0.43	−0.19	−0.32
TRo/RC	0.11	−0.56	0.42	0.18	0.68	0.72	−0.04	−0.59	0.19	0.17	0.52	0.10	0.06
ETo/RC	−0.30	0.07	0.02	−0.33	0.42	−0.03	−0.44	0.65	0.86 *	0.99 ***	0.61	0.70	0.63
PI_abs_	−0.36	−0.16	0.14	−0.52	0.51	0.07	−0.58	0.62	0.73	0.89 **	0.79	0.48	0.42
PI_total_	−0.11	0.08	0.39	−0.72	0.21	−0.27	−0.85 *	0.57	0.40	0.66	0.53	0.11	0.02
RWC	−0.41	−0.11	−0.14	−0.32	0.51	0.04	−0.35	0.75	0.88 *	0.96 **	0.71	0.71	0.68
Chl *a*	−0.49	−0.32	−0.09	−0.27	0.65	0.32	−0.26	0.56	0.86 *	0.91 **	0.85 *	0.69	0.67
Chl *b*	0.41	−0.18	0.13	0.47	−0.21	0.24	0.44	−0.88 *	−0.76	−0.9 **	−0.5	−0.61	−0.57
Tot Chl	0.29	−0.29	0.08	0.64	0.43	0.54	0.40	−0.69	0.14	−0.03	0.03	0.24	0.22

* *p* ≤ 0.05, ** *p* ≤ 0.01, *** *p* ≤ 0.001. TSS = total soluble sugars, FAA = Free amino acids, Asx = Arginine + Aspartate, Ala = Alanine, Glx = Glutamate + Glutamine, Arg = Arginine, Gly = Glycine, Pro = Proline, PRI = photochemical reflectance index, NDVI = normalized difference vegetative index, SPAD = structure intensive pigment index, CCI = chlorophyll content index, Fv/Fm = maximum photosystem II (PSII) quantum yield ratio of variable to maximum fluorescence, ABS/RC = energy absorbed per reaction center, DIo/RC = energy dissipated as heat per reaction center. TRo/RC = trapped energy per reaction center, ETo/RC = the flux of electrons transferred from quinone (QA) to plastoquinone (PQ) per active PSII per reaction center, PI_abs_ = performance index absorbance, PI_total_ =total performance index, RWC = relative water content, Chl *a* = chlorophyll *a*, Chl *b* = chlorophyll *b*, Tot Chl = total chlorophyll content.

## Data Availability

The original contributions presented in this study are included in the article/Supplementary Material. Further inquiries can be directed to the corresponding author.

## Supplementary Materials

The following supporting information can be downloaded at: https://www.mdpi.com/article/10.3390/plants15131943/s1. Figure S1: Soybean growth stages (VE–R8) and approximate days after planting. Table S1. Three-way ANOVA on the effects of selenium soil drench on amino acids, soluble sugars and photosynthetic efficiency of vegetable-soybean under drought stress at flowering stage. Table S2. Three-way ANOVA on the effects of selenium soil drench on amino acids, soluble sugars and photosynthetic efficiency of vegetable-soybean under drought stress at pod-filling stage. (See Ref. [65]).

## List of Abbreviations

**Abbreviation**	**Definition**
WHC	Water-holding capacity
C	Cultivar
Se	Selenium
SE	Standard error
FW	Fresh weight
PSI	Photosystem I
PSII	Photosystem II
QA	Quinone
PQ	Plastoquinone
LSD	Least significant difference
ANOVA	Analysis of variance
RWC	Relative water content
FAA	Free amino acid
TSS	Total soluble sugars
PRI	Photochemical reflectance index
NDVI	Normalized difference vegetative index
CCI	Chlorophyll content Index
SPAD	Structure intensive pigment index
Fv/Fm	Maximum photosystem II (PSII) quantum yield ratio of variable to maximum fluorescence
ABS/RC	Energy absorbed per reaction center
DIo/RC	Energy dissipated as heat per reaction center
TRo/RC	Trapped energy per reaction center
ETo/RC	The flux of electrons transferred from quinone (QA) to plastoquinone (PQ) per active PSII per reaction center
PI_abs_	Performance index absorbance
PI_total_	Total performance index
Chl *a*	Chlorophyll *a*
Chl *b*	Chlorophyll *b*
Tot Chl	Total chlorophyll content
Asx	Arginine + Aspartate
Glx	Glutamate + Glutamine
Ala	Alanine
Arg	Arginine
Gly	Glycine
Pro	Proline

## References

[1] Hussain M., Farooq S., Hasan W., Ul-Allah S., Tanveer M., Farooq M., Nawaz A. (2018). Drought stress in sunflower: Physiological effects and its management through breeding and agronomic alternatives. Agric. Water Manag..

[2] Shrestha S., Mahat J., Shrestha J., Madhav K.C., Paudel K. (2022). Influence of high-temperature stress on rice growth and development. A review. Heliyon.

[3] Fisher M., Abate T., Lunduka R.W., Asnake W., Alemayehu Y., Madulu R.B. (2015). Drought tolerant maize for farmer adaptation to drought in sub-Saharan Africa: Determinants of adoption in eastern and southern Africa. Clim. Change.

[4] Pagliarini R.F., Marinho J.P., Molinari M.D.C., Marcolino-Gomes J., Caranhoto A.L.H., Marin S.R.R., Oliveira M.C.N., Foloni J.S.S., Melo C.L.P., Kidokoro S. (2021). Overexpression of full-length and partial DREB2A enhances soybean drought tolerance. Agron. Sci. Biotechnol..

[5] Zhang F., Li X., Wu Q., Lu P., Kang Q., Zhao M., Wang A., Dong Q., Sun M., Yang Z. (2022). Selenium Application Enhances the Accumulation of Flavones and Anthocyanins in Bread Wheat (*Triticum aestivum* L.) Grains. J. Agric. Food Chem..

[6] Zeeshan M., Wang X., Salam A., Wu H., Li S., Zhu S., Chang J., Chen X., Zhang Z., Zhang P. (2024). Selenium Nanoparticles Boost the Drought Stress Response of Soybean by Enhancing Pigment Accumulation, Oxidative Stress Management and Ultrastructural Integrity. Agronomy.

[7] Sallam A., Alqudah A.M., Dawood M.F.A., Baenziger P.S., Börner A. (2019). Drought Stress Tolerance in Wheat and Barley: Advances in Physiology, Breeding and Genetics Research. Int. J. Mol. Sci..

[8] Miao Q., Ma X., Liu Y., Wang Y. (2015). Effects of drought stress on growth and yield of Tulipa edulis. Sci. Hortic..

[9] Van der Merwe R., Tyawana S., Van der Merwe J., Mwenye O. (2018). Evaluation of drought tolerance indices in vegetable-type soybean. Soc. Cienc. Galicia.

[10] Sousaraei R., Ghassemi-Golezani K., Farhoudi R. (2021). Photosynthetic responses of tomato seedlings to drought stress. Photosynthetica.

[11] Yan W., Lu Y., Guo L., Liu Y., Li M., Zhang B., Zhang B., Zhang L., Qin D., Huo J. (2024). Effects of Drought Stress on Photosynthesis and Chlorophyll Fluorescence in Blue Honeysuckle. Plants.

[12] Sadak M.S., Bakhoum G.S. (2022). Selenium-induced modulations in growth, productivity and physiochemical responses to water deficiency in Quinoa (*Chenopodium quinoa*) grown in sandy soil. Biocatal. Agric. Biotechnol..

[13] Hlahla J.M., Mafa M.S., van der Merwe R., Alexander O., Duvenhage M.M., Kemp G., Moloi M.J. (2022). The Photosynthetic Efficiency and Carbohydrates Responses of Six Edamame (*Glycine max*. L. Merrill) Cultivars under Drought Stress. Plants.

[14] Krasavina M.S., Burmistrova N.A., Raldugina G.N., Ahmad P., Rasool S. (2014). The Role of Carbohydrates in Plant Resistance to Abiotic Stresses. Emerging Technologies and Management of Crop Stress Tolerance: Biological Techniques.

[15] Bolouri-Moghaddam M.R., Le Roy K., Xiang L., Rolland F., Van den Ende W. (2010). Sugar signalling and antioxidant network connections in plant cells. FEBS J..

[16] Moloi M.J., van der Merwe R. (2021). Drought tolerance responses in vegetable-type soybean involve a network of biochemical mechanisms at flowering and pod-filling stages. Plants.

[17] Keunen E., Peshev D., Vangronsveld J., Van Den Ende W., Cuypers A. (2013). Plant sugars are crucial players in the oxidative challenge during abiotic stress: Extending the traditional concept. Plant Cell Environ..

[18] Cui Y.N., Yan S.J., Zhang Y.N., Wang R., Song L.L., Ma Y., Guo H., Yang P.Z. (2024). Physiological, Metabolome and Gene Expression Analyses Reveal the Accumulation and Biosynthesis Pathways of Soluble Sugars and Amino Acids in Sweet Sorghum under Osmotic Stresses. Int. J. Mol. Sci..

[19] Suprasanna P., Nikalje G.C., Rai A.N., Iqbal N., Nazar R., Khan N.A. (2016). Osmolyte accumulation and implications in plant abiotic stress tolerance. Osmolytes and Plants Acclimation to Changing Environment: Emerging Omics Technologies.

[20] Baqir H.A., Zeboon N.H., Al-Behadili A.A.J. (2019). The role and importance of amino acids within plants: A review. Plant Arch..

[21] Brotosudarmo T., Limantara L., Dwi Chandra R., Heriyanto H. (2018). Chloroplast Pigments: Structure, Function, Assembly and Characterization. Plant Growth and Regulation: Alterations to Sustain Unfavorable Conditions.

[22] Dien D.C., Mochizuki T., Yamakawa T. (2019). Effect of various drought stresses and subsequent recovery on proline, total soluble sugar and starch metabolisms in Rice (*Oryza sativa* L.) varieties. Plant Prod. Sci..

[23] Baskaran A., Muruganandam A. (2017). Enhancing Role of Proline in Scavenging Free Radicals by Antioxidative Defence System during Stress in Black Gram and Cluster Bean. Int. J. Pharm. Sci. Rev. Res..

[24] Hlahla J.M., Mafa M.S., van der Merwe R., Moloi M.J. (2024). Exploring edamame survival mechanisms under combined drought and heat stress: Photosynthesis efficiency and carbohydrate accumulation. Plant Stress.

[25] Conti V., Cantini C., Romi M., Cesare M.M., Parrotta L., Del Duca S., Cai G. (2022). Distinct Tomato Cultivars Are Characterized by a Differential Pattern of Biochemical Responses to Drought Stress. Int. J. Mol. Sci..

[26] Brooks R.R., Farago M.E. (1998). Plants that hyperaccumulate heavy metals. Plant and the Chemical Elements: Biochemistry, Uptake, Tolerance and Toxicity.

[27] Aggarwal M., Sharma S., Kaur N., Pathania D., Bhandhari K., Kaushal N., Kaur R., Singh K., Srivastava A., Nayyar H. (2011). Exogenous proline application reduces phytotoxic effects of selenium by minimising oxidative stress and improves growth in bean (*Phaseolus vulgaris* L.) seedlings. Biol. Trace Elem. Res..

[28] Hajiboland R., Sadeghzadeh N., Ebrahimi N., Sadeghzadeh B., Mohammadi S.A. (2015). Influence of selenium in drought-stressed wheat plants under greenhouse and field conditions. Acta Agric. Slov..

[29] Hajiboland R., Sadeghzadeh N., Sadeghzadeh B. (2014). Effect of Se application on photosynthesis, osmolytes and water relations in two durum wheat (*Triticum durum* L.) genotypes under drought stress. Acta Agric. Slov..

[30] Zahedi S.M., Moharrami F., Sarikhani S., Padervand M. (2020). Selenium and silica nanostructure-based recovery of strawberry plants subjected to drought stress. Sci. Rep..

[31] Khalofah A., Migdadi H., El-Harty E. (2021). Antioxidant Enzymatic Activities and Growth Response of Quinoa (Chenopodium quinoa Willd) to Exogenous Selenium Application. Plants.

[32] Semida W.M., Abd El-Mageed T.A., Abdelkhalik A., Hemida K.A., Abdurrahman H.A., Howladar S.M., Leilah A.A.A., Rady M.O.A. (2021). Selenium Modulates Antioxidant Activity, Osmoprotectants, and Photosynthetic Efficiency of Onion under Saline Soil Conditions. Agronomy.

[33] Moloi M.J., Khoza B.M. (2022). The Effect of Selenium Foliar Application on the Physiological Responses of Edamame under Different Water Treatments. Agronomy.

[34] Sekhurwane M., Tóth B., Moloi M.J. (2025). Effectiveness of Soil, Foliar, and Seed Selenium Applications in Modulating Physio-Biochemical, and Yield Responses to Drought Stress in Vegetable Soybean (*Glycine max* L. Merrill). Plants.

[35] Ježek P., Hlušek J., Lošák T., Jůzl M., Elzner P., Kráčmar S., Buňka F., Martensson A. (2011). Effect of foliar application of selenium on the content of selected amino acids in potato tubers (*Solanum tuberosum* L.). Plant Soil Environ..

[36] Sors T.G., Ellis D.R., Salt D.E. (2005). Selenium uptake, translocation, assimilation and metabolic fate in plants. Photosynth. Res..

[37] Krasensky J., Jonak C. (2012). Drought, salt, and temperature stress-induced metabolic rearrangements and regulatory networks. J. Exp. Bot..

[38] Zeier J. (2013). New insights into the regulation of plant immunity by amino acid metabolic pathways. Plant Cell Environ..

[39] Wang Y., Zhu Q., Wang Z.W., Wang J.P., Wang Z., Yu F.Y., Zhang L.H. (2024). Effects of foliar application of amino acid-chelated selenite on photosynthetic characteristics of peanut (*Arachis hypogaea* L.) leaves at the podding stage. Plant Soil Environ..

[40] Carillo P., Mastrolonardo G., Nacca F., Fuggi A. (2005). Nitrate reductase in durum wheat seedlings as affected by nitrate nutrition and salinity. Funct. Plant Biol..

[41] Annunziata M.G., Ciarmiello L.F., Woodrow P., Maximova E., Fuggi A., Carillo P. (2017). Durum wheat roots adapt to salinity by remodelling the cellular content of nitrogen metabolites and sucrose. Front. Plant Sci..

[42] Ali Q., Athar H., Haider M.H., Shahid S., Aslam N., Shehzad F., Naseem J., Ashraf R., Ali A., Hussain S.M., Hasanuzzaman M., Fujita M., Oku H., Islam M.T. (2019). Role of Amino Acids in Improving Abiotic Stress Tolerance to Plants. Plant Tolerance to Environmental Stress.

[43] Yadav R., Saini R., Adhikary A., Kumar S. (2022). Unravelling cross priming induced heat stress, combinatorial heat and drought stress response in contrasting chickpea varieties. Plant Physiol. Biochem..

[44] Romanenko O.M., Babenko L.M. (2024). Amino acids in regulation of abiotic stress tolerance in cereal crops: A review. Cereal Res. Commun..

[45] Elkelish A., Soliman M.H., Alhaithloul H.A., El-Esawi M.A. (2019). Selenium protects wheat seedlings against salt stress-mediated oxidative damage by up-regulating antioxidants and osmolytes metabolism. Plant Physiol. Biochem..

[46] Planchet E., Limami A.M., D’Mello J.P.F. (2015). Amino Acid Synthesis under Abiotic Stress. Amino Acids in Higher Plants.

[47] Chilimba A.D.C., Young S.D., Black C.R., Meacham M.C., Lammel J., Broadley M.R. (2012). Agronomic biofortification of maize with selenium (Se) in Malawi. Field Crops Res..

[48] Khan N., Ali S., Zandi P., Mehmood A., Ullah S., Ikram M., Ismail I., Shahid M.A., Babar A. (2020). Role of sugars, amino acids and organic acids in improving plant abiotic stress tolerance. Pak. J. Bot..

[49] Cunha M.L.O., de Oliveira L.C.A., Mendes N.A.C., Silva V.M., Vicente E.F., dos Reis A.R. (2023). Selenium Increases Photosynthetic Pigments, Flavonoid Biosynthesis, Nodulation, and Growth of Soybean Plants (*Glycine max* L.). J. Soil Sci. Plant Nutr..

[50] Fan S., Wu H., Gong H., Guo J. (2022). The salicylic acid mediates selenium-induced tolerance to drought stress in tomato plants. Sci. Hortic..

[51] Delorge I., Janiak M., Carpentier S., Van Dijck P. (2014). Fine tuning of trehalose biosynthesis and hydrolysis as a tool for controlling plant metabolism and development. Plant Sci..

[52] Lunn J.E., Delorge I., Figuero C.M., Van Dijck P., Stitt M. (2014). Trehalose metabolism in plants. Plant J..

[53] Magen S., Daniel S., Weiss S., Factor D.J., Mursalimov S., Soroka Y., Michaeli S., Avin-Wittenberg T. (2024). Raffinose induces autophagy to promote plant growth. bioRxiv.

[54] Malik J.A., Kumar S., Thakur P., Sharma S., Kaur N., Kaur R., Pathania D., Bhandhari K., Kaushal N., Singh K. (2011). Promotion of growth in mungbean (*Phaseolus aureus* Roxb.) by selenium is associated with stimulation of carbohydrate metabolism. Biol. Trace Elem. Res..

[55] Du Z.Y., Wang Q., Chen L.J., Zhao X.Y. (2020). Selenium improves photosynthetic efficiency and sucrose metabolism in soybean under drought stress. Plant Physiol. Rep..

[56] Garousi F., Veres S., Bódi É., Várallyay S., Kovács B. (2016). Assessment and comparison of selenium-enriched maize with sodium selenite and sodium selenate. Acta Agr. Debreceniensis.

[57] Kalaji H.M., Schansker G., Ladle R.J., Goltsev V., Bosa K., Allakhverdiev S.I., Brestic M., Bussotti F., Calatayud A., Dąbrowski P. (2014). Frequently asked questions about in vivo chlorophyll fluorescence: Practical issues. Photosynth. Res..

[58] Stirbet A., Lazár D., Kromdijk J., Govindjee G. (2018). Chlorophyll *a* fluorescence induction: Can just a one-second measurement be used to quantify abiotic stress responses?. Photosynthetica.

[59] Muhammed I., Shalmani A., Ali M., Yang Q.H., Ahmad H., Li F.B. (2020). Mechanisms regulating the dynamics of photosynthesis under abiotic stresses. Front. Plant Sci..

[60] Su S., Zhou Y., Qin J., Yao W., Ma Z. (2010). Optimization of the method for chlorophyll extraction in aquatic plants. J. Freshw. Ecol..

[61] Gonzáles L., Gonzáles-Vilar M., Reigosa Roger M.J. (2001). Determination of relative water content. Handbook of Plant Ecophysiology Techniques.

[62] Correia I., Nunes A., Duarte I.F., Barros A., Delgadillo I. (2005). Sorghum fermentation followed by spectroscopic techniques. Food Chem..

[63] Højrup P. (2024). Analysis of Polypeptides by Amino Acid Analysis. Methods Mol. Biol..

[64] Irigoyen J.J., Einerich D.W., Sánchez-Díaz M. (1992). Water stress induced changes in concentrations of proline and total soluble sugars in modulated alfalfa (*Medicago sativa*) plants. Physiol. Plant..

[65] Fehr W.R., Caviness C.E., Burmood D.T., Pennington J.S. (1971). Stage of Development Descriptions for Soybeans, *Glycine Max* (L.) Merrill 1. Crop Sci..

