# Supplementary material for: Insights into Selenium-Modulated Amino Acids and Carbohydrates as Osmolytes Linked to Photosynthetic Efficiency in Drought-Stressed Edamame
Source: Plants (Basel). 2026 Jun 24;15(13):1943. doi: 10.3390/plants15131943 (PMC13363813; doi:10.3390/plants15131943)
Supplement: Supplementary file 1 [file plants-15-01943-s001.zip › plants-4353357-supplementary.pdf]

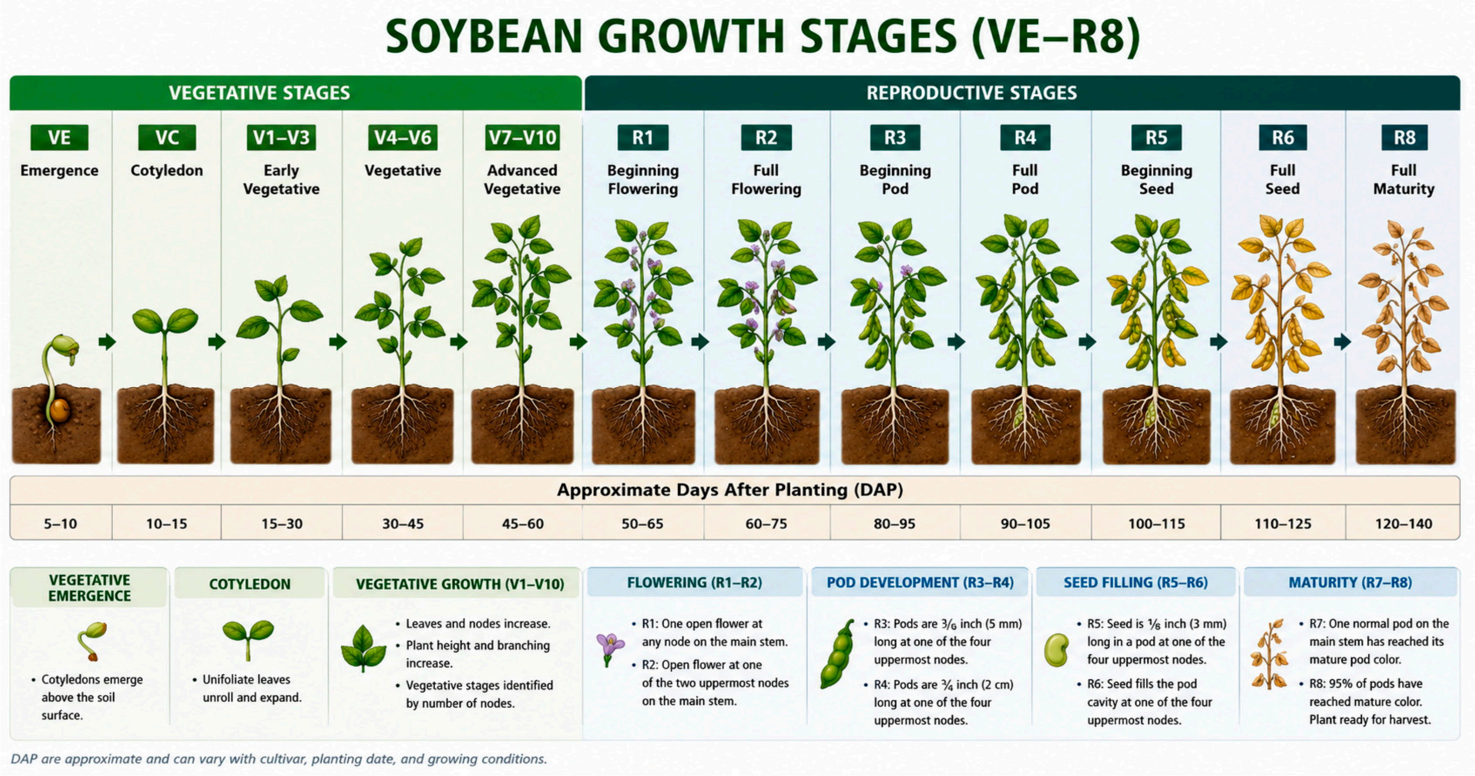

Figure S1: Soybean growth stages (VE–R8) and approximate days after planting.

Image Adapted from Fehr and Caviness (1).

Table S1. represents the main effects of cultivar (C), water level (W) and selenium (Se), their two-way interactions (C x Se), (W x Se) as well as their three-way interactions (C x W x Se) on amino acids, soluble sugars and photosynthetic parameters of vegetable-soybean cultivars (UVE14 and UVE17) at the flowering and pod-filling stages (ANOVA). At the flowering stage, cultivars exhibited distinct responses to selenium soil application under different water regimes, as evidenced by significant cultivar x selenium x water-level interactions for NDVI, fructose, glucose ($p \leq 0.05$), and for CCI, SPAD, Fv/Fm, ABS/RC, PI_{abs}, Chl *a*, Asx, Glx and TSS ($p \leq 0.001$). At pod-filling, significant three-way interactions were also observed for CCI, SPAD, PI_{total}, RWC, TSS and Asx ($p \leq 0.001$), NDVI and raffinose ($p \leq 0.01$) and Glx ($p \leq 0.05$).

Among traits without three-way effects, significant cultivar x selenium interactions occurred at flowering for PRI ($p \leq 0.05$), PI_{total}, Chl *b*, Asx and Tot Chl ($p \leq 0.001$), glucose and sucrose ($p \leq 0.01$). At the pod-filling stage, the interaction was significant for PRI, fructose ($p \leq 0.01$), PI_{abs}, Chl *a*, Chl *b* ($p \leq 0.001$) and Tot Chl ($p \leq 0.05$). Furthermore, at flowering, the interaction between water treatment and selenium differed significantly for glucose ($p \leq 0.01$), PI_{total}, FAA and Asx ($p \leq 0.001$). At pod-filling stage, the interaction was significant for ABS/RC, DIo/RC, ETo/RC ($p \leq 0.01$), Chl *a* ($p \leq 0.001$), TRo/RC, and glucose ($p \leq 0.05$).

For traits that showed neither three-way nor two-way interactions, only the main effects were interpreted. Cultivars were significant for DIo/RC, TRo/RC, ETo/RC, RWC, raffinose, glycine, Asx ($p \leq 0.001$), and alanine ($p \leq 0.01$) at flowering. At the pod-filling stage, significant differences were

observed for ABS/RC ($p \leq 0.001$), DIo/RC, ETo/RC ($p \leq 0.01$), and TRo/RC ($p \leq 0.05$). Water treatments were significant for DIo/RC, TRo/RC, ETo/RC, RWC, sucrose, glycine, Asx ($p \leq 0.001$), raffinose ($p \leq 0.01$), and alanine ($p \leq 0.05$) at flowering. At the pod-filling stage, the water levels showed significant differences in PRI ($p \leq 0.01$), Fv/Fm ($p \leq 0.001$), and fructose ($p \leq 0.05$).

Table S1. Three-way ANOVA on the effects of selenium soil drench on amino acids, soluble sugars and photosynthetic efficiency of vegetable-soybean under drought stress at flowering stage.

**Flowering**						
	**Cultivar (C)**	**Water (W)**	**Selenium (Se)**	**C x Se**	**W x Se**	**C x W x Se**
PRI	0.004***	0.0013***	0.000	0.00006*	0.000*	0.000
NDVI	0.787***	0.735***	0.000**	0.00010*	0.000**	0.000*
CCI	187.74***	102.16***	1.346***	1.06**	0.236	7.08***
SPAD	972.68***	718.28***	0.59	2.784**	0.31	8.711***
Fv/Fm	2.986***	3.144***	0.0017**	0.0065***	0.002***	0.0119***
ABS/RC	3.73***	0.66***	0.045***	0.0429***	0.085***	0.089***
DIo/RC	886.62***	907.26***	0.0774	0.193	0.1423	0.4194
TRo/RC	285.56***	304.49***	0.0031	0.146	0.0187	0.2723
ETo/RC	118.11***	115.97***	0.0006	0.0085	0.0101	0.0380
PI_{abs}	9.93***	51.998***	0.0208	0.665**	2.452***	4.976***
PI_{total}	167.09***	121.78***	1.65***	1.139***	2.778***	0.1918
RWC	8190.6***	11288.8***	0.10	0.02	0.93	0.27
Chl *a*	1210***	1171.74***	2.16***	1.63***	2.45***	1.606***
Chl *b*	0.29***	0.438***	0.017*	0.239***	0.0001	0.0013
Tot Chl	0.24***	4.4105***	0.22***	0.181***	0.000	0.000
TSS	2.21***	1.365***	0.03***	0.138***	0.002	0.0971***
Glucose	0.698**	0.94***	1.04***	0.358**	0.371**	0.055
Sucrose	0.482**	0.542***	0.161	0.348**	0.030	0.0038
Fructose	10008***	10016***	0.898*	0.958**	0.927**	0.847*
Trehalose	0.171	0.167	0.028	0.237	0.016	0.0401
Raffinose	2.94***	2.703**	0.002	0.021	0.004	0.027
FAA	1.41***	3.60***	0.069**	0.00045	0.109***	0.0058
Arg	0.030	0.284***	0.00035	0.036	0.078	0.02
Gly	435.6***	432.25	3.090	3.96	4.095	2.786
Asx	0.1715	0.07836	0.16915	0.01905	0.04543	0.7198***
Glx	0.81698	0.1744	0.24284	0.11422	0.12011	0.87***
Ala	0.082**	0.068*	0.0129	0.052	0.023	0.0394
Pro	0.099	0.311*	0.036	0.01	0.000	0.0405

*p ≤ 0.05, **p≤ 0.01,***p≤ 0.001. TSS = total soluble sugars, FAA = Free amino acids, Arg = Arginine, Gly = Glycine, Asx = Asparagine + Aspartate, Glx = Glutamate + Glutamine, Ala = Alanine, Pro = Proline, PRI = Photochemical reflectance index, chlorophyll index, NDVI = Normalized difference vegetative index, SPAD = Structure intensive pigment index, CCI = Chlorophyll content Index, SPAD = structure intensive pigment index, Fv/Fm = maximum photosystem II (PSII) quantum yield ratio of variable to maximum fluorescence, ABS/RC = energy absorbed per reaction centre, DIo/RC = energy dissipated as heat per reaction centre. TRo/RC = trapped energy per reaction centre, ETo/RC = the flux of electrons transferred from quinone (QA) to plastoquinone (PQ) per active PSII per reaction centre, PI_{abs} = performance index absorbance, PI_{total} = total performance index, RWC = relative water content, Chl *a* = chlorophyll a, Chl *b* = chlorophyll b, Tot Chl = total chlorophyll content. Values represent the mean squares.

Table S2. Three-way ANOVA on the effects of selenium soil drench on amino acids, soluble sugars and photosynthetic efficiency of vegetable-soybean under drought stress at pod-filling stage.

	Pod-filling					
	**Cultivar (C)**	**Water (W)**	**Selenium (Se)**	**C x Se**	**W x Se**	**C x W x Se**
PRI	0.0000*	0.00043**	0.000	0.00**	0.000	0.00
NDVI	0.000***	0.0000	0.000	0.000	0.000**	0.0002**
CCI	0.824	17.47***	0.095	0.003	0.079	14.506***
SPAD	1.441**	24.59***	2.67***	0.40	0.02	22.36***
Fv/Fm	0.00027	0.006***	0.00045	0.000	0.000	0.000
ABS/RC	0.219***	2.100***	0.237***	0.013	0.023**	0.005
DIo/RC	0.0066**	0.184***	0.024***	0.000	0.0058**	0.002
TRo/RC	0.138*	0.673***	0.117*	0.003	0.111*	0.061
ETo/RC	0.0054**	0.0061**	0.0017	0.000	0.0260**	0.000
PI_{abs}	9.798***	24.46***	11.15***	1.1356***	0.1988	0.0115
PI_{total}	4.699***	13.35***	3.473***	0.7579***	0.08353	0.838***
RWC	0.3	18.875**	0.9	49.490***	5.4207*	29.70***
Chl *a*	0.161***	0.962***	0.082***	0.042***	0.031***	0.0029
Chl *b*	0.131***	0.700***	0.0049	0.1846***	0.00486	0.0006
Tot Chl	0.2789**	1.1757***	0.317***	0.110*	0.0079	0.0142
TSS	0.0016	0.00003	0.011**	0.031***	0.015***	0.0135***
Glucose	0.00093	0.014**	0.00175	0.00103	0.008*	0.00031
Sucrose	0.0579	0.0068	0.0058	0.0697	0.0121	0.0028
Fructose	0.008	0.0136*	0.0160*	0.02**	0.0051	0.0053
Trehalose	0.034	0.0014	0.0147	0.0080	0.0585	0.1021
Raffinose	0.006*	0.0033	0.007*	0.0042	0.0058*	0.0082**
FAA	0.0261	0.000	0.003	0.0251	0.0088	0.00011
Arg	0.0261	0.0238	0.024	0.025	0.0277	0.026
Gly	0.0078	0.0083	0.083	0.0042	0.0037	0.116
Asx	0.074***	0.110219	0.073***	0.079***	0.097***	0.080***
Glx	0.013172	0.024313	0.0805	0.022	0.028646	0.11*
Ala	0.0125	0.0127	0.0102	0.0139	0.0149	0.0145
Pro	0.0087	0.0089	0.0050	0.0097	0.0120	0.0104

*p ≤ 0.05, **p≤ 0.01,***p≤ 0.001. TSS = total soluble sugars, FAA = Free amino acids, Arg = Arginine, Gly = Glycine, Asx = Asparagine + Aspartate, Glx = Glutamate + Glutamine, Ala = Alanine, Pro = Proline, PRI = Photochemical reflectance index, chlorophyll index, NDVI = Normalized difference vegetative index, SPAD = Structure intensive pigment index, CCI = Chlorophyll content Index, SPAD = structure intensive pigment index, Fv/Fm = maximum photosystem II (PSII) quantum yield ratio of variable to maximum

fluorescence, ABS/RC = energy absorbed per reaction centre, DIo/RC = energy dissipated as heat per reaction centre. TRo/RC = trapped energy per reaction centre, ETo/RC = the flux of electrons transferred from quinone (QA) to plastoquinone (PQ) per active PSII per reaction centre, PI_{abs} = performance index absorbance, PI_{total} = total performance index, RWC = relative water content, Chl *a* = chlorophyll a, Chl *b* = chlorophyll b, Tot Chl = total chlorophyll content. Values represent the mean squares.

References

1. Fehr, W.R., Caviness, C.E., Burmood, D.T. and Pennington, J.S. (1971). Stage of Development Descriptions for Soybeans, Glycine Max (L.) Merrill 1. *Crop Science*, 11(6), pp.929–931. doi:https://doi.org/10.2135/cropsci1971.0011183x001100060051x.
